# Adenylyl Cyclase 8 in Dorsal CA1 Neurons Prevents Depressive‐Like Behaviors by Maintaining Neuronal Excitability and Glutamatergic Neurotransmission Through TIP39‐PTH2R Signaling

**DOI:** 10.1002/advs.202512170

**Published:** 2025-12-12

**Authors:** Zi‐Jie Liu, Jia‐Rui Bi, Zong‐Yan Yu, Meng Tian, Zhi‐Yue Chen, Ran Wei, Miao‐Miao Wang, Hai‐Wei Zha, Yu‐Qing Zhang, Hong‐Jing Wang, Bang‐You Qiang, Shuang‐Shuang Sun, Xiao‐Juan Zhu, Wen‐Bing Chen, Dong Sun

**Affiliations:** ^1^ National Engineering Laboratory for AIDS Vaccine School of Life Sciences Jilin University Changchun China; ^2^ The Affiliated TCM Hospital School of Basic Medical Sciences Guangzhou Medical University Guangzhou China; ^3^ Key Laboratory of Neurogenetics and Channelopathies of Guangdong Province and the Ministry of Education of China Guangzhou Medical University Guangzhou China; ^4^ Key Laboratory of Molecular Epigenetics Ministry of Education Institute of Genetics and Cytology Northeast Normal University Changchun China; ^5^ Key Laboratory for Molecular Enzymology and Engineering The Ministry of Education School of Life Sciences Jilin University Changchun China

**Keywords:** *Adcy*8, depression, Glutamatergic neurotransmission, neuronal excitability, Pth2r

## Abstract

Depression, a common neuropsychiatric disorder, profoundly disrupts individuals' daily lives. Although the pathogenesis of depression is intensively investigated for decades, its underlying mechanisms remain elusive. Here, dysfunctional adenylyl cyclase 8 (*Adcy8*) is identified as a critical risk factor for the development of depression. *Adcy8* expression is selectively decreased in the hippocampus, but not in the cortex, thalamus, and hypothalamus, of mice exposed to chronic stress. *Adcy8* conditional knockout (CKO) in excitatory neurons, particularly dorsal CA1 (dCA1) neurons, resulted in pronounced depressive‐like behaviors. Depletion of *Adcy8* in dCA1 neurons reduces neuronal excitability and glutamatergic neurotransmission. Further mechanistic studies reveal a remarkable inhibition of the mitogen‐activated protein kinase (MAPK) signaling pathway by *Adcy8* CKO, which downregulates parathyroid hormone 2 receptor (PTH2R) level in the hippocampus. Knocking down *Pth2r* with AAV‐shRNA duplicates the impairments in neuronal excitability, glutamatergic neurotransmission and depressive‐like behaviors. In contrast, overexpression of PTH2R in *Adcy8* CKO hippocampus rescues these deficits. Chronic infusion of TIP39, the endogenous ligand for PTH2R, into the hippocampus also alleviates depressive‐like behaviors of *Adcy8* CKO mice. Taken together, these results uncover critical roles of *Adcy8* in suppressing depressive‐like behaviors, likely by maintaining the excitability and glutamatergic neurotransmission of dCA1 neurons through TIP39‐PTH2R signaling pathway.

## Graphical abstract

Depression, a prevalent neuropsychiatric disorder with unclear pathogenesis, involves dysfunctional adenylyl cyclase 8 (*Adcy8*) as a key risk factor. Chronic stress selectively reduces *Adcy8* expression in the dorsal CA1 (dCA1) neurons. Depletion of *Adcy8* in dCA1 excitatory neurons induces depressive‐like behaviors by impairing neuronal excitability and glutamatergic neurotransmission, which is mediated by inhibiting MAPK signaling and decreasing PTH2R levels.

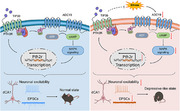

## 1 Introduction

Depression is affected by both genetic and environmental factors.^[^ ^1^ ^]^ Patients with depression typically display a persistent feeling of sadness, hopelessness and loss of interest, which leads to noticeable disruptions in daily activities. Over the past several decades, therapeutic strategies for depression have undergone extensive investigation, including pharmacological interventions, psychotherapy and electroconvulsive methods. Accumulating evidence suggests that interventions targeting specific neurological pathways, including the monoamine neurotransmitter system, may yield a certain antidepressant effect.^[^ ^2^ ^]^ Nevertheless, a significant proportion of patients with depression exhibit treatment resistance.^[^ ^3^ ^]^ Thus, it is of considerable interest to fully understand the underlying mechanisms of depression pathogenesis, which may contribute to the development of diagnostic and therapeutic strategies and eventually improve brain health.

Chronic stress, a well‐known risk factor for neuropsychiatric disorders, is increasingly found to be associated with the onset of depression.^[^ ^4^ ^]^ Chronic exposure to stress induces the hyperactivity of hypothalamic‐pituitary‐adrenal (HPA) axis, resulting in excessive production of cortisol in humans or corticosterone in rodents.^[^ ^5^ ^]^ Prolonged elevation of cortisol/corticosterone level impairs normal physiological functions across multiple brain regions, including hippocampus, amygdala and prefrontal cortex, which are involved in mood regulation and emotional processing.^[^ ^6^, ^7^ ^]^ Although pharmacological interventions targeting the HPA axis, or those using monoamine neurotransmitters and ketamine confer a promising therapeutic approach for depression, they may also have limited clinical efficacy and trigger several side effects.^[^ ^8^, ^9^, ^10^ ^]^ Thus, elucidating the detailed link between chronic stress and depression is meaningful for combating this disorder.

As reported, a growing body of evidence has demonstrated that hippocampus serves as a key target of chronic stress. Clinical studies showed that chronic stress led to a significant decrease in hippocampal volume, correlating with the atrophy phenotype observed in depressed patients.^[^ ^11^, ^12^, ^13^ ^]^ In addition, chronic stress has been reported to reduce hippocampal neurogenesis, alter neuronal electrophysiological properties and disrupt depression‐related signaling pathways in the hippocampus of animal models.^[^ ^14^, ^15^, ^16^ ^]^ Several specific genes within the hippocampus are also associated with stress‐related depression.^[^ ^17^, ^18^, ^19^ ^]^ However, hippocampal molecular alterations that might play a causal role in the development of depression remain elusive. Adenylyl cyclases (ADCYs) are unique enzymes that catalyze the genesis of cyclic adenosine monophosphate (cAMP) from Adenosine triphosphate (ATP).^[^ ^20^ ^]^ The activation of ADCYs has been found to correlate with antidepressant responses in patients,^[^ ^21^ ^]^ and genome‐wide association study (GWAS) have suggested roles of *ADCY3*, *ADCY7* and *ADCY8* in depression.^[^ ^22^ ^]^ In addition, downregulated cAMP signaling has been frequently observed in depressed patients and animals exposed to chronic stress.^[^ ^23^, ^24^ ^]^ However, whether and how hippocampal ADCYs contribute to depression warrants investigations.

Here, we provide evidence that *Adcy8* in dorsal CA1 (dCA1) neurons plays a critical role in regulating mouse depressive‐like behaviors. Chronic stress selectively decreased *Adcy8* expression in the dCA1 neurons. Mice with *Adcy8* conditional knockout (CKO) in CaMKII^+^ neurons (Adcy8^CaMKII^‐CKO) exhibited pronounced depressive‐like behaviors. Further analyses revealed reduced dCA1 calcium signals in response to stress, along with decreased neuronal excitability and glutamatergic neurotransmission in dCA1 neurons of Adcy8^CaMKII^‐CKO mice. RNA‐sequencing screening for molecular mechanism identified that the parathyroid hormone 2 receptor (PTH2R), a class B G‐protein coupled receptor (GPCR), was significantly decreased in Adcy8^CaMKII^‐CKO hippocampus. Knockdown of hippocampal *Pth2r* also inhibited neuronal excitability and decreased glutamatergic neurotransmission in dCA1, and induced depressive‐like behaviors. Finally, overexpression of PTH2R in Adcy8^CaMKII^‐CKO hippocampus restored the excitability and neurotransmission of dCA1 neurons, and alleviated the depressive‐like behaviors. Moreover, chronic infusion of TIP39, an endogenous ligand for PTH2R, also produces an anti‐depressant effect in Adcy8^CaMKII^‐CKO mice. Taken together, these results demonstrate an unrecognized role of *Adcy8* in regulating neuronal excitability, glutamatergic neurotransmission, and depressive‐like behaviors, likely through modulating the TIP39‐PTH2R signaling pathway.

## 2 Result

### 2.1 Chronic Stress‐Induced Downregulation of *Adcy8* in dCA1 Neurons Serves as a Risk Factor for Depressive‐Like Behaviors in Mice

**Figure 1:**
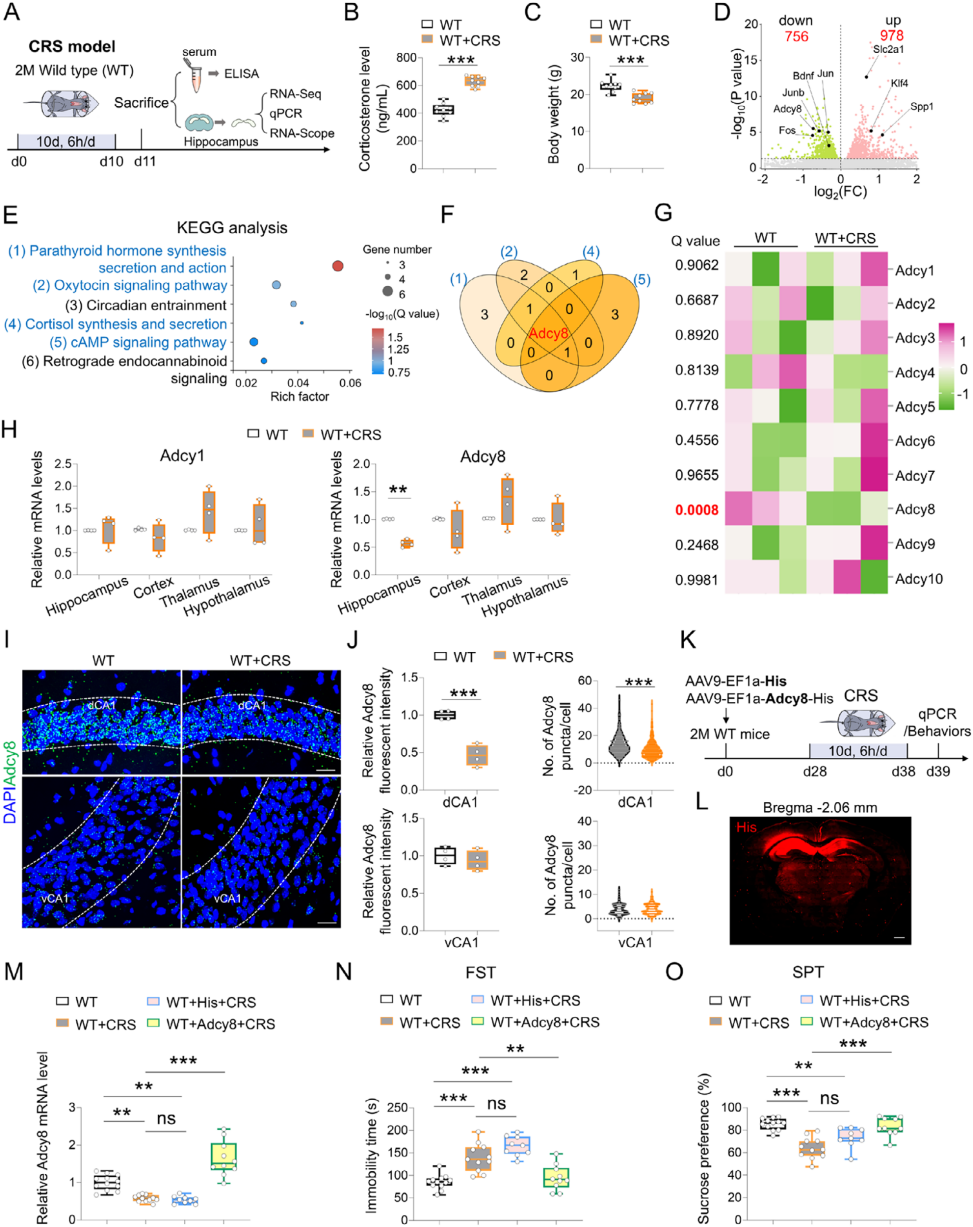
CRS selectively decreased *Adcy8* expression in the hippocampus and increasing *Adcy8* expression reversed the depressive‐like behaviors of CRS treated mice. A) Schematic diagram of experimental design for ELISA, RNA‐seq, qPCR, and RNA‐scope in WT and WT+CRS mice. B) ELISA analysis of corticosterone in the plasma of WT and WT+CRS mice. *n* = 9 mice for each group. Student's *t*‐test. *p* < 0.001. C) Body weight analysis of WT and WT+CRS mice. *n* = 9 mice for each group. Student's *t*‐test. *p* < 0.001. D) Volcano plots of differentially expressed genes in the WT+CRS hippocampus as compared to WT hippocampus. E) KEGG analysis of the downregulated genes in D. F) Venn diagram of selected signaling pathways in E (blue). G) Heatmap of *Adcy*1‐10 in the hippocampus of WT and WT+CRS mice. H) qPCR analyses of *Adcy1* and *Adcy8* mRNA levels in the hippocampus, cortex, thalamus and hypothalamus. *n* = 4 mice for each group. For *Adcy1* (WT versus WT+CRS): Student's t test for hippocampus, *p* = 0.7798; cortex, *p* = 0.3474; Mann‐Whitney U test for thalamus, *p* = 0.3429; hypothalamus, *p* > 0.9999. For *Adcy8* (WT versus WT+CRS): Student's *t*‐test for hippocampus, *p* = 0.005; thalamus, *p* = 0.2278; hypothalamus, *p* = 0.9941; Mann‐Whitney U test for cortex, *p* = 0.3429; I) representative RNA‐scope images of *Adcy8* in dCA1 and vCA1 subregions of WT and WT+CRS mice. Scale bar = 20 µm. J) Quantifications of the data in I, the fluorescent intensity of *Adcy8* mRNA, and the number of *Adcy8* puncta surrounding cell nucleus in WT and WT+CRS mice. *n* = 4 mice for each group. Student's *t*‐test for *Adcy8* fluorescent intensity comparison. *p* < 0.001; Mann‐Whitney U test for *Adcy8* puncta comparison. *p* < 0.001. K) Schematic diagram of experimental design for virus injection in WT mice, and 10 days of CRS followed by qPCR analysis and behavioral tests. L) Immunostaining of His tag in the brain to indicate the appropriate injection site of viruses. Scale bar = 100 µm. M) qPCR analyses of *Adcy8* mRNA levels in the hippocampus of each group of mice. *n* = 12 mice in WT group; n = 11 mice in WT+CRS group; n = 9 mice in WT+His+CRS group and n = 10 mice in WT+Adcy8+CRS group. One‐way ANOVA followed by Tukey's multiple comparisons test. F_(3, 38)_ = 39.03, *p* < 0.001. WT versus WT+CRS, *p* = 0.0019; WT versus WT+His+CRS, *p* = 0.0013; WT+CRS versus WT+His+CRS, *p* = 0.9881; WT+CRS versus WT+Adcy8+CRS, *p* < 0.001. N) Quantifications of immobility time in the FST of WT mice (*n* = 12), WT+CRS mice (*n* = 11), WT+His+CRS mice (*n* = 9) and WT+Adcy8+CRS mice (*n* = 10). One‐way ANOVA followed by Tukey's multiple comparisons test. F_(3, 38)_ = 23.34, *p* < 0.001. WT versus WT+CRS, *p* < 0.001; WT versus WT+His+CRS, *p* < 0.001; WT+CRS versus WT+His+CRS, *p* = 0.1158; WT+CRS versus WT+Adcy8+CRS, *p* = 0.0012. O) Quantifications of sucrose preference in the SPT of WT mice (*n* = 12), WT+CRS mice (*n* = 11), WT+His+CRS mice (*n* = 9) and WT+Adcy8+CRS mice (*n* = 10). One‐way ANOVA followed by Tukey's multiple comparisons test. F_(3, 38)_ = 16.80, *p* < 0.001. WT versus WT+CRS, *p* < 0.001; WT versus WT+His+CRS, *p* = 0.0068; WT+CRS versus WT+His+CRS, *p* = 0.0646; WT+CRS versus WT+Adcy8+CRS, *p* < 0.001. Data in B, C, H, J, M, N, and O are presented as median with interquartile range, whiskers are the minimum and maximum. **p < 0.01, ***p < 0.001.

To investigate the potential risk factors by which chronic stress contributes to depression, we subjected wild‐type (WT) mice to a chronic restraint stress (CRS) paradigm, a well‐established mouse model of stress. Mice were exposed to CRS for 10 consecutive days with 6 h restraint per day. Following this treatment, RNA‐sequencing and biochemical analyses were performed in both control and CRS‐exposed mice at indicated time (**Figure** **1**A). As shown in Figure 1B,C, 10 days of CRS significantly increased the plasma level of corticosterone and reduced body weight as compared to control mice, suggesting the elevated stress state of WT+CRS mice. Furthermore, CRS induced remarkable depressive‐like behaviors in WT mice, as WT+CRS mice spent less time struggling and more time floating in the forced swim test (FST), and displayed less sucrose solution consumption in the sucrose preference test (SPT) than those in control mice (Figure S1A,B, Supporting Information), suggesting the depressive‐like symptoms (e.g., despair and anhedonia) after exposure to CRS. Additionally, a decrease of center exploration in the open field test (OFT) and a significant reduction of duration time on open arms in the elevated plus maze test (EPMT) were observed in WT+CRS mice as compared with those in control mice (Figure S1C,D, Supporting Information), suggesting increased anxiety‐like behaviors by CRS stimuli. These behavioral changes thus support the view that CRS impaired emotional regulation in mice.

Next, we explored the effect of CRS on hippocampal transcriptomic alterations using RNA‐sequencing analysis. As shown in Figure 1D, 756 downregulated genes and 978 upregulated genes were found in WT+CRS hippocampus as compared to control hippocampus, including previously identified stress‐responsive genes (e.g., *Fos*, *Junb*, and *Bdnf*) and neurological diseases related genes (e.g., *Spp1*, *Klf4*, and *Slc2a1*).^[^ ^25^, ^26^, ^27^, ^28^, ^29^, ^30^ ^]^ Kyoto Encyclopedia of Genes and Genomes (KEGG) analysis of dysregulated genes showed a pronounced association with mood regulatory pathways, including parathyroid hormone synthesis, secretion and action, oxytocin signaling pathway, cortisol synthesis and secretion, and cAMP signaling pathway (Figure 1E). Notably, *Adcy8*, an overlapped gene in these pathways, is significantly decreased in the hippocampus of WT+CRS mice as compared to control mice, whereas its expression remains unaltered in the cortex, thalamus and hypothalamus (Figure 1F–H). Meanwhile, *Adcy1*, along with other *Adcy* genes involved in cAMP production, showed few changes following CRS stimuli (Figure 1G,H). By comparison, although 1 day of acute restraint stress (ARS) also increased serum corticosterone levels and induced hundreds of dysregulated genes in the hippocampus compared to control mice, it did not alter *Adcy8* mRNA levels (Figure S2A–D, Supporting Information). KEGG enrichment analysis of these ARS‐induced dysregulated genes further identified several distinct signaling pathways compared to those induced by CRS, including gap junction, valine, leucine and isoleucine degradation, and endocytosis (Figure S2E, Supporting Information). Collectively, these results indicate that *Adcy8* expression in the hippocampus is specifically reduced by CRS, rather than by ARS.

Next, to further map the brain regions with decreased *Adcy8* mRNAs, RNA‐scope analysis was performed by incubating *Adcy8* probes with brain sections from WT and WT+CRS mice. The fluorescent intensity of *Adcy8* was much lower in dCA1 regions of WT+CRS mice than that in control mice, and the number of *Adcy8* mRNA puncta per dCA1 neuron was substantially decreased following CRS exposure (Figure 1I,J). In contrast, no such decreases in *Adcy8* mRNAs were observed in other hippocampal subregions of WT+CRS mice, including ventral CA1 (vCA1), dorsal dentate gyrus (dDG), dorsal CA2 (dCA2), dorsal CA3 (dCA3), ventral dentate gyrus (vDG) and ventral CA3 (vCA3), as compared with those in control mice (Figure 1I,J; Figure S3, Supporting Information). These results thus suggest that CRS selectively decreased *Adcy8* expression in dCA1 neurons. Next, to further verify this phenotype, we utilized chronic social defeat stress (CSDS), another widely used mouse stress model, to treat WT mice (Figure S4A, Supporting Information). As shown in Figure S4B–D (Supporting Information), WT+CSDS mice were divided into two subgroups: resilient mice displaying minimal changes in social and depressive‐like behaviors as compared to WT mice, and susceptible mice exhibiting deficits in social behaviors and obvious depressive‐like behaviors. Subsequently, *Adcy8* expression in the dCA1 region was assessed in WT, WT+CSDS (resilient) and WT+CSDS (susceptible) mice by RNA‐scope analysis. Interestingly, 10 days of CSDS significantly reduced *Adcy8* mRNA levels only in WT+CSDS (susceptible) mice, but not in WT+CSDS (resilient) mice (Figure S4E,F, Supporting Information). Together, these results indicate that *Adcy8* expression in dCA1 neurons is downregulated in response to chronic stress, suggesting that such a decrease may contribute to stress‐associated depressive‐like behaviors.

Next, to determine whether increasing *Adcy8* expression in the dCA1 neurons reverses the depressive‐like behaviors of WT+CRS mice, we bilaterally injected either AAV9‐EF1a‐His (control virus) or AAV9‐EF1a‐Adcy8‐His (virus for *Adcy8* overexpression) into the dCA1 regions of WT mice. 4 weeks later, both groups of mice were subjected to CRS for 10 days. Subsequently, qPCR analysis and behavioral tests were conducted to assess *Adcy8* expression and mouse depressive‐like behaviors, respectively (Figure 1K,L). As expected, *Adcy8* mRNAs were largely increased in the dorsal hippocampus of WT+Adcy8+CRS mice as compared to WT+CRS mice, whereas WT+His+CRS mice still showed a significant decrease in *Adcy8* expression as compared to WT mice, at a similar level with that in WT+CRS mice (Figure 1M), suggesting that AAV9‐EF1a‐Adcy8‐His, rather than AAV9‐EF1a‐His, successfully increased *Adcy8* expression in CRS exposed mice. Notably, compared to WT+CRS mice, WT+Adcy8+CRS exhibited reduced immobility time in the FST and increased sucrose preference ratio in the SPT (Figure 1N,O), suggesting that overexpression of *Adcy8* decreased depressive‐like behaviors of WT+CRS mice. In contrast, no such anti‐depression effect was observed in WT+CRS mice injected with control virus, because the behavioral performance of WT+His+CRS mice was comparable to WT+CRS mice (Figure 1N,O). Collectively, these findings indicate that increasing *Adcy8* expression in the dCA1 neurons is sufficient to reverse the depressive‐like behaviors of WT+CRS mice, thus supporting a causal role of *Adcy8* downregulation in chronic stress induced depressive‐like behaviors.

### 2.2 Knocking Out *Adcy8* in Hippocampal Excitatory Neurons Promotes Depressive‐Like Behaviors

**Figure 2:**
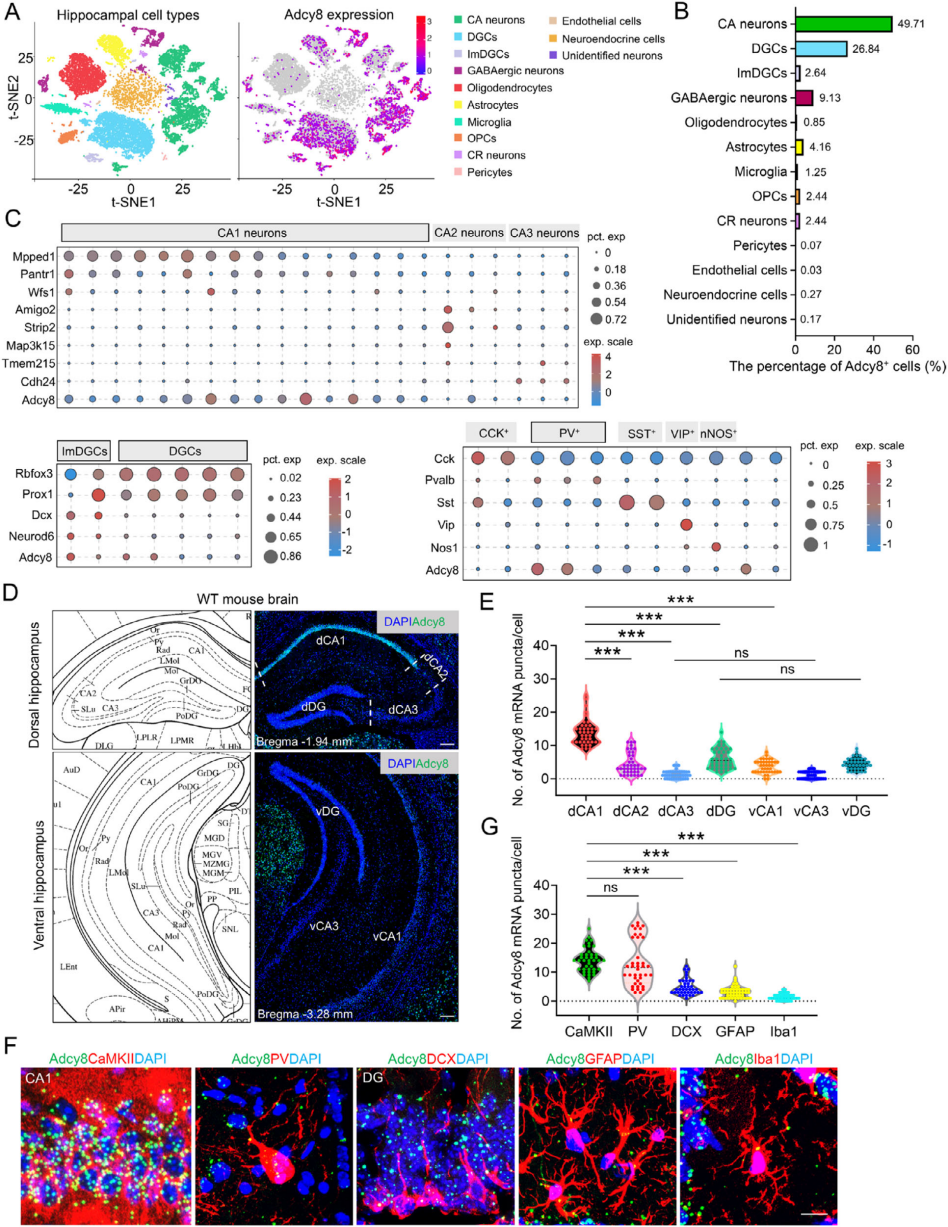
*Adcy8* is highly expressed in hippocampal neurons. A) t‐SNE map shows *Adcy8* expression in the hippocampus. B) Quantification of the percentage of *Adcy8* ^+^ cells over total cells. C) Bubble charts show *Adcy8* expression in the CA neurons, DG neurons and GABAergic neurons. D) Representative RNA‐scope image of *Adcy8* mRNA in the dorsal and ventral hippocampus. Scale bar = 200 µm. E) Quantification of the data in D), the number of *Adcy8* puncta surrounding cell nucleus of dCA1, dCA2, dCA3, dDG, vCA1, vCA3, and vDG neurons. *n* = 5 slices. One‐way ANOVA followed by Tukey's multiple comparisons test. F_(6, 273)_ = 120.1, *p*<0.001. dCA1 versus dCA2, *p* < 0.001; dCA1 versus dCA3, *p* < 0.001; dCA1 versus dDG, *p* < 0.001; dCA1 versus vCA1, *p* < 0.001; dCA3 versus vCA3, *p* > 0.9999; dDG versus vDG, *p* = 0.0831. F) Co‐staining analyses of *Adcy8* (green) with different cell markers: CaMKII for excitatory neurons; PV for inhibitory neurons; DCX for immature DGCs (ImDGCs); GFAP for astrocyte and Iba1 for microglia. Scale bar = 10 µm. G) Quantifications of the data in F), the number of *Adcy8* puncta surrounding cell nucleus of different cell types. *n* = 5 slices. One‐way ANOVA followed by Tukey's multiple comparisons test. F_(4, 201)_ = 76.77, *p* < 0.001. CaMKII versus PV, *p* = 0.5957; CaMKII versus DCX, *p* < 0.001; CaMKII versus GFAP, *p* < 0.001; CaMKII versus Iba1, *p* < 0.001. Data in E and G) are presented as violin plot with all points. ***p < 0.001.

To investigate the roles of hippocampal *Adcy8* in regulating emotional behaviors, we first examined the expression of *Adcy8* in mouse hippocampus by taking advantage of snRNA‐sequencing analysis. As shown in **Figure** **2**A,B, a large proportion of *Adcy8^+^* cells were neurons, including CA pyramidal neurons, dentate granule cells (DGCs) and GABAergic neurons. In contrast, only a small number of *Adcy8^+^* cells were astrocytes, immature DGCs (ImDGCs), oligodendrocyte progenitor cells (OPCs) and Cajal‐Retzius (CR) neurons. Meanwhile, *Adcy8* mRNA was barely detected in oligodendrocytes, microglia, pericytes and endothelial cells. The re‐cluster analysis of distinct cell types also demonstrated that *Adcy8* mRNAs were enriched in CA1 pyramidal neurons, ImDGCs and DGCs, and parvalbumin‐positive (PV^+^) interneurons (Figure 2C). Next, we performed RNA‐scope analysis to further verify the distribution of *Adcy8* mRNAs in the hippocampus. As shown in Figure 2D,E, *Adcy8* was found to exhibit remarkably high expression in the dCA1 subregion, moderate expression in the dDG, partial expression in the dCA2 close to dCA1, and minimal mRNA levels in the dCA3. By comparison, *Adcy8* expression in the vCA1 was significantly lower than that in dCA1, while its mRNA levels in the vCA3 and vDG were comparable to those in their respective dorsal counterparts. Furthermore, co‐staining of *Adcy8* probes with different cell type markers revealed much higher *Adcy8* expression in CaMKII^+^ dCA1 pyramidal neurons and PV^+^ interneurons when compared to DCX^+^ ImDGCs and GFAP^+^ astrocytes, and few *Adcy8* mRNAs were detected in Iba1^+^ microglial cells (Figure 2F,G). Of note, CRS specifically decreased *Adcy8* mRNAs in dCA1 CaMKII^+^ neurons, but not in vCA1 CaMKII^+^ neurons (Figure S5A,B, Supporting Information). Additionally, *Adcy8* expression in PV^+^ inhibitory neurons remained unaltered by CRS in both the dCA1 and vCA1 subregions (Figure S5A,C, Supporting Information).

**Figure 3:**
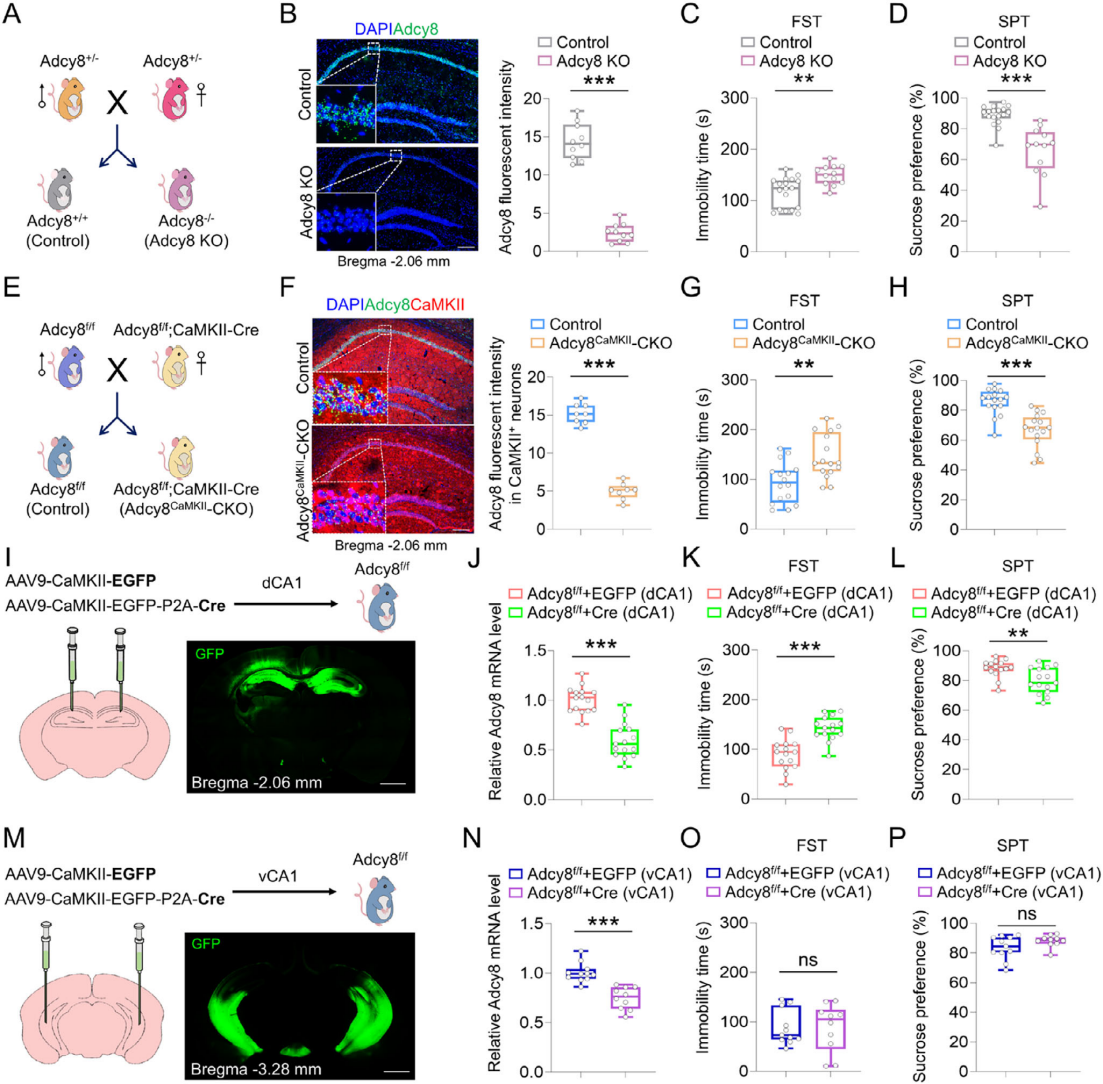
*Adcy8* CKO in hippocampal dCA1 neurons lead to depressive‐like behaviors in mice. A) Schematic diagram of breeding strategy to generate Adcy8^−/−^ (Adcy8 KO) mice. Adcy8^+/+^ mice are control. B) RNA‐scope analysis of *Adcy8* mRNAs in the dorsal hippocampus of control and Adcy8 KO mice. High magnification of the selected region in dCA1 was shown beside each image. *n* = 10 slices from 4 mice in each group. Scale bar = 200 µm. Student's *t*‐test. *p* < 0.001. C) Quantifications of immobility time in the FST of control (*n* = 18) and Adcy8 KO (*n* = 12) mice. Mann‐Whitney U test. *p* = 0.0019. D) Quantifications of sucrose preference in the SPT of control (*n* = 18) and Adcy8 KO (*n* = 12) mice. Mann‐Whitney U test. *p* < 0.001. E) Schematic diagram of breeding strategy to generate Adcy8^CaMKII^‐CKO mice. Adcy8^f/f^ mice are control. F) RNA‐scope analysis of Adcy8 mRNAs in the dorsal hippocampus of control and Adcy8^CaMKII^‐CKO mice. High magnification of the selected region in dCA1 was shown beside each image. *n* = 8 slices from 4 mice in each group. Scale bar = 200 µm. Student's *t*‐test. *p* < 0.001. G) Quantifications of immobility time in the FST of control (*n* = 16) and Adcy8^CaMKII^‐CKO (*n* = 16) mice. Student's *t*‐test. *p* = 0.0014. H) Quantifications of sucrose preference in the SPT of control (*n* = 16) and Adcy8^CaMKII^‐CKO (*n* = 16) mice. Student's *t*‐test. *p* < 0.001. I) Schematic diagram of experimental design for virus injection in Adcy8^f/f^ mice. GFP signaling indicates the appropriate injection site in dCA1 regions. Control (AAV9‐CaMKII‐EGFP) viruses express GFP; Cre (AAV9‐CaMKII‐EGFP‐P2A‐Cre) viruses express GFP and a nuclear‐localized Cre recombinase. Scale bar = 300 µm. J) qPCR analysis of *Adcy8* mRNA levels in the hippocampus of Adcy8^f/f^+GFP (*n* = 15) and Adcy8^f/f^+Cre (*n* = 15) mice. Student's *t*‐test. *p* < 0.001. K) Quantifications of immobility time in the FST of Adcy8^f/f^+GFP (*n* = 15) and Adcy8^f/f^+Cre (*n* = 15) mice. Student's *t*‐test. *p* < 0.001. L) Quantifications of sucrose preference in the SPT of Adcy8^f/f^+GFP (*n* = 15) and Adcy8^f/f^+Cre (*n* = 15) mice. Student's *t*‐test. *p* = 0.005. M) Schematic diagram of experimental design for virus injection in Adcy8^f/f^ mice. GFP signaling indicates the appropriate injection site in vCA1 regions. Control (AAV9‐CaMKII‐EGFP) viruses express GFP; Cre (AAV9‐CaMKII‐EGFP‐P2A‐Cre) viruses express GFP and a nuclear‐localized Cre recombinase. Scale bar = 300 µm. N) qPCR analysis of *Adcy8* mRNA levels in the hippocampus of Adcy8^f/f^+GFP (*n* = 11) and Adcy8^f/f^+Cre (*n* = 10) mice. Student's *t*‐test. *p* < 0.001. O) Quantifications of immobility time in the FST of Adcy8^f/f^+GFP (*n* = 11) and Adcy8^f/f^+Cre (*n* = 10) mice. Student's *t*‐test. *p* = 0.9817. P) Quantifications of sucrose preference in the SPT of Adcy8^f/f^+GFP (*n* = 11) and Adcy8^f/f^+Cre (*n* = 10) mice. Student's *t*‐test. *p* = 0.1236. Data in B–D, F–H, J–L, and N–P are presented as median with interquartile range, whiskers are the minimum and maximum. **p < 0.01, ***p < 0.001.

Next, to determine whether *Adcy8* deficiency contributes to the development of neuropsychiatric disorders in mice, we generated whole‐body *Adcy8* KO mice and their littermate controls by *Adcy8* heterozygous × heterozygous mating strategy (**Figure** **3**A). As shown in Figure 3B, RNA‐scope analysis in the hippocampus showed that *Adcy8* mRNAs were substantially depleted in *Adcy8* KO mice. Subsequent behavioral tests revealed that *Adcy8* KO mice displayed more immobility time in the FST and less sucrose preference in the SPT than control mice (Figure 3C,D), as well as exhibited decreased time exploring the center area in the OFT and reduced duration time and entries to open arms in the EPMT as compared to control mice (Figure S6A,B, Supporting Information), suggesting an essential role of *Adcy8* in regulating mouse depressive‐ and anxiety‐like behaviors.

Notice that global KO mouse model limits the investigation of tissue/cell‐specific roles of *Adcy8*. To address this issue, based on the expression pattern of *Adcy8* in hippocampus, we conditionally knocked out (referred to as CKO) *Adcy8* gene by crossing Adcy8^f/f^ mice with CaMKII‐Cre or PV‐Cre mice to generate Adcy8^CaMKII^‐CKO or Adcy8^PV^‐CKO mice (Figure 3E; Figure S7A, Supporting Information). Thus, *Adcy8* was CKO in CaMKII‐Cre^+^ excitatory neurons and PV‐Cre^+^ inhibitory neurons, respectively. As shown in Figure 3F, *Adcy8* mRNAs were largely depleted in the dCA1 neurons of Adcy8^CaMKII^‐CKO mice as compared with those in Adcy8^f/f^ (referred to as control) mice. Intriguingly, behavioral tests showed that Adcy8^CaMKII^‐CKO mice exhibited pronounced depressive‐like behaviors in the FST and SPT (Figure 3G,H), but little to no differences in the OFT and EPMT as compared to control mice (Figure S6C,D, Supporting Information). In contrast, Adcy8^PV^‐CKO mice with *Adcy8* CKO in PV^+^ interneurons displayed comparable levels with control mice in the FST, SPT, OFT and EPMT (Figure S7B–F, Supporting Information). Thus, these results highlight the key role of *Adcy8* in CaMKII‐Cre^+^ excitatory neurons rather than in PV^+^ interneurons to regulate mouse depressive‐like behaviors.

As reported,^[^ ^31^ ^]^ CaMKII‐Cre was largely detected in excitatory neurons of the whole forebrain. To determine whether *Adcy8* in hippocampal excitatory neurons is critical, AAV9‐CaMKII‐EGFP (referred to as EGFP) or AAV9‐CaMKII‐EGFP‐P2A‐Cre (referred to as Cre), which drive the GFP or Cre expression under the control of CaMKII promoter, were bilaterally injected into the dCA1 regions of Adcy8^f/f^ mice (Figure 3I). The level of *Adcy8* mRNAs was significantly decreased in Adcy8^f/f^+Cre (dCA1) hippocampus as compared with that in Adcy8^f/f^+GFP (dCA1) hippocampus (Figure 3J). Consistent with the observation in Adcy8^CaMKII^‐CKO mice, local depletion of *Adcy8* in dCA1 neurons again induced depressive‐like behaviors in the FST and SPT (Figure 3K,L). However, comparable performance in the FST and SPT was observed between Adcy8^f/f^+GFP (vCA1) and Adcy8^f/f^+Cre (vCA1) mice, in which *Adcy8* was specifically CKO in vCA1 neurons by Cre‐expressing viruses (Figure 3M–P). Collectively, these results demonstrate that *Adcy8* CKO in dCA1 excitatory neurons leads to depressive‐like behaviors.

### 2.3 Decreased dCA1 Neuron Activation Induced by Stress in Adcy8^CaMKII^‐CKO Mice

**Figure 4:**
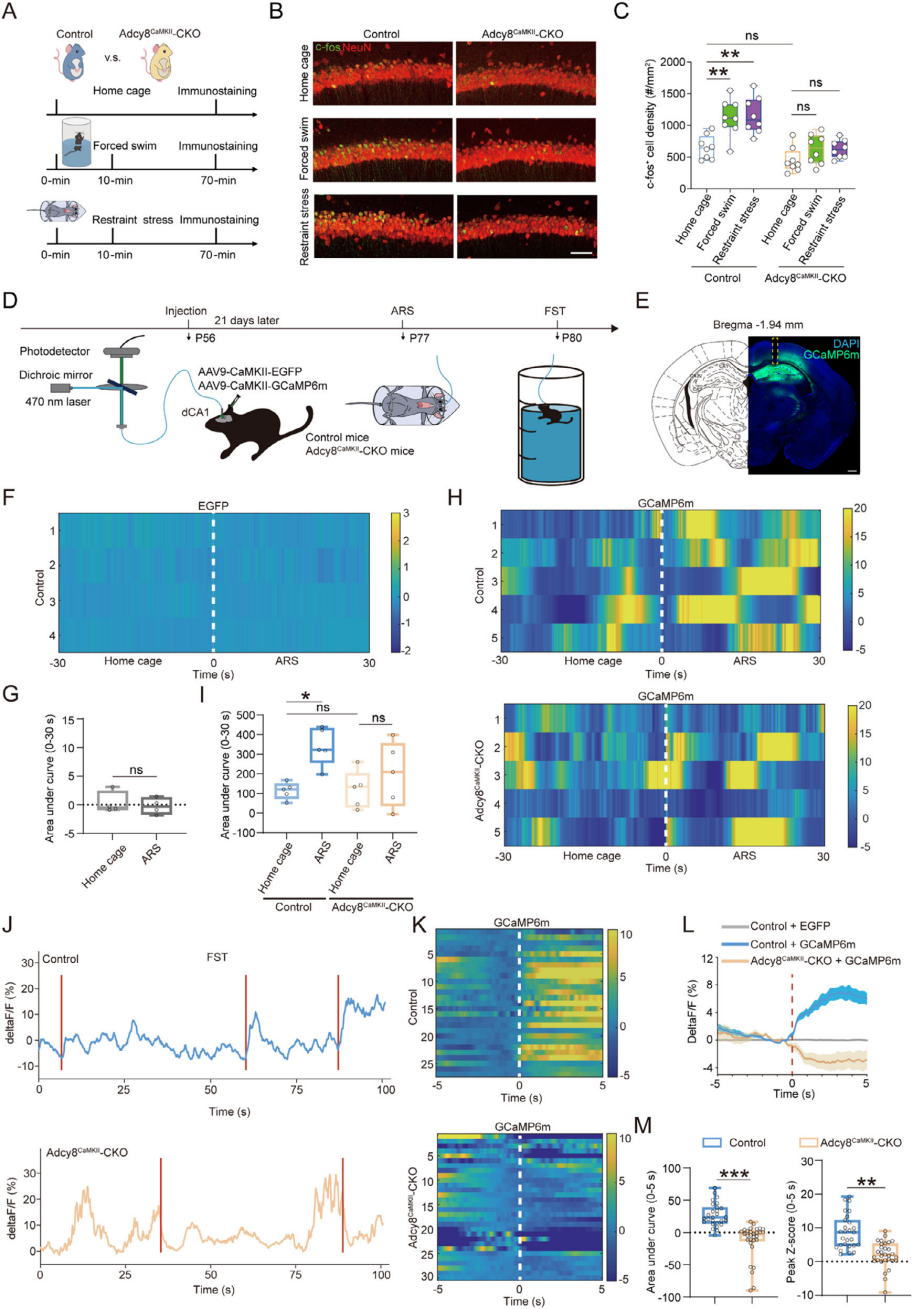
Decreased neuronal activity in the hippocampus of Adcy8^CaMKII^‐CKO mice. A) Schematic diagram of experimental design for FST and ARS, and subsequent immunostaining analysis. B) Representative images of c‐fos (green) and NeuN (red) in the dCA1 regions of control and Adcy8^CaMKII^‐CKO mice with or without FST and ARS stimuli. Scale bar = 50 µm. C) Quantification of the number of c‐fos^+^ cells in B. *n* = 8 slices from 4 mice in each group. Two‐way ANOVA followed by Tukey's multiple comparisons test. F_(5, 42)_ = 13.00, *p* < 0.001. For control mice: home cage versus forced swim, *p* = 0.0024; home cage versus restraint stress, *p* = 0.0011; For Adcy8^CaMKII^‐CKO mice: home cage versus forced swim, *p* = 0.6595; home cage versus restraint stress, *p* = 0.6598; For home cage: control versus Adcy8^CaMKII^‐CKO, p = 0.5071. D) Schematic diagram of ARS and FST with simultaneous fiber photometry in the dCA1 region of control and Adcy8^CaMKII^‐CKO mice. E) Representative image showing AAV‐delivered GCaMP6m expression and optical fiber placement in the dCA1 region. Scale bar = 100 µm. F) Heatmaps of GFP signals in the dCA1 neurons of control mice during home cage and ARS state. G) Quantification of averaged area under curve (AUC) in F to indicate GFP signal changes by ARS. *n* = 4 mice for each genotype. Mann‐Whitney *U* test, *p* = 0.8857. H) Heatmaps of calcium transients in the dCA1 neurons of control and Adcy8^CaMKII^‐CKO mice during home cage and ARS state. I) Quantification of AUC in H to indicate GCaMP6m signal changes by ARS. *n* = 5 mice for each genotype. Two‐way ANOVA followed by Tukey's multiple comparisons test. Interaction: F_(1, 16)_ = 2.286, *p* = 0.1500; treatment factor: F_(1, 16)_ = 9.768, *p* = 0.0065; genotype factor: F_(1, 16)_ = 1.942, *p* = 0.1825. For home cage: control versus Adcy8^CaMKII^‐CKO, *p* = 0.9998; For control mice: home cage versus ARS, p = 0.022; For Adcy8^CaMKII^‐CKO mice: home cage versus ARS, p = 0.6707. J) Representative traces of GCaMP6m signal recorded. Colored stripes (red) indicate the bouts when mice start to struggle from resting state. K) Heatmaps of calcium transients evoked by struggling in control and Adcy8^CaMKII^‐CKO mice. 27 bouts from control and 30 bouts from Adcy8^CaMKII^‐CKO mice. *n* = 6 mice for each genotype. L) Averaged calcium transients in the dCA1 region of control and Adcy8^CaMKII^‐CKO mice. Shaded areas around means indicated error bars. (M) AUC of bouts and peak Z‐score of GCaMP6m signals of bouts. Mann‐Whitney *U* test for AUC, *p* < 0.001; Student's *t*‐test for peak Z‐score, *p* < 0.001. Data in C, G, I, and M are presented as median with interquartile range, whiskers are the minimum and maximum. **p < 0.01, ***p < 0.001. ns = no significance.

To understand the effect of *Adcy8*‐deficiency on neuronal functions, we initiated our investigation by measuring the activities of dCA1 neurons in both control and Adcy8^CaMKII^‐CKO mice. As shown in **Figure** **4**A, each group of mice were exposed to FST or ARS for 10 min, which served as an aversive stressor. In contrast, mice staying in the home cage are in the basal condition. 1 hour later, their brain samples were collected and subjected to immunostaining analysis using antibody against c‐fos, a well‐known marker for neuron activation.^[^ ^32^ ^]^ Interestingly, the number of c‐fos^+^ cells in the dCA1 region of control mice was similar with that in Adcy8^CaMKII^‐CKO mice under basal condition (Figure 4B,C). However, when mice were exposed to FST or ARS, remarkable increases in c‐fos expression were observed in the dCA1 neurons of control mice, whereas no such increases were detected in Adcy8^CaMKII^‐CKO mice (Figure 4B,C). These findings suggest that FST or ARS selectively activated dCA1 neurons in control mice, but failed to induce the same response in Adcy8^CaMKII^‐CKO mice.

Next, to figure out how dCA1 neurons respond to stressful stimuli in awake mice, we examined the dynamic calcium signals by using in vivo calcium recording technique. As shown in Figure 4D,E, AAV9‐CaMKII‐GCaMP6m was injected into the dCA1 region to express GCaMP6m as the calcium indicator at postnatal 56 days; mice injected with AAV9‐CaMKII‐EGFP were controls. 3 weeks later, the fluorescent signal of GFP or GCaMP6m were recorded in control mice via an optic fiber placed above dCA1 neurons. As expected, minimal changes in GFP fluorescent intensity were detected in these mice during home cage activity and ARS state, suggesting a stable GFP signaling in this assay and excluding the non‐specific fluorescence interference (Figure 4F,G). Interestingly, there was no significant difference in GCaMP6m signals between control and Adcy8^CaMKII^‐CKO mice during spontaneous activity in the home cage. However, the GCaMP6m fluorescent signal in dCA1 neurons of control mice increased rapidly when they were restrained in the tubes, whereas ARS did not significantly enhance calcium signals in dCA1 neurons of Adcy8^CaMKII^‐CKO mice (Figure 4H,I). Moreover, in line with literature report,^[^ ^33^ ^]^ FST induced mice to swim vigorously to escape the aversive environment. And meanwhile, the fluorescent intensity of GCaMP6m in the dCA1 neurons of control mice increased rapidly (Figure 4J). Of note is that in vivo calcium recordings only revealed increases in GCaMP6m signals when control mice, but not Adcy8^CaMKII^‐CKO mice, start to struggle from floating state in the FST (Figure 4J–L; Videos S1 and S2, Supporting Information). In addition, control mice exhibited an 85.2% probability of struggling behavior accompanied by calcium transients in dCA1 neurons, whereas this probability was substantially reduced to only 13.3% in Adcy8^CaMKII^‐CKO mice (Figure S8, Supporting Information). Both the total and the highest fluorescent intensity of GCaMP6m in a 5 s time window following the onset of struggling behavior were also reduced in Adcy8^CaMKII^‐CKO mice as compared to control mice (Figure 4M). Thus, it seems that struggling behaviors in the FST are closely associated with calcium transients in dCA1 neurons; however, *Adcy8* CKO in CaMKII^+^ neurons disrupts this correlation. Taken together, these results indicate that depletion of *Adcy8* abolished the increase of calcium signals in dCA1 neurons induced by ARS or FST, suggesting an indispensable role of *Adcy8* in dCA1 neuron activation after stress stimuli.

### 2.4 Reduced Neuronal Excitability and Impaired Glutamatergic Neurotransmission in the dCA1 Neurons of Adcy8^CaMKII^‐CKO Mice

**Figure 5:**
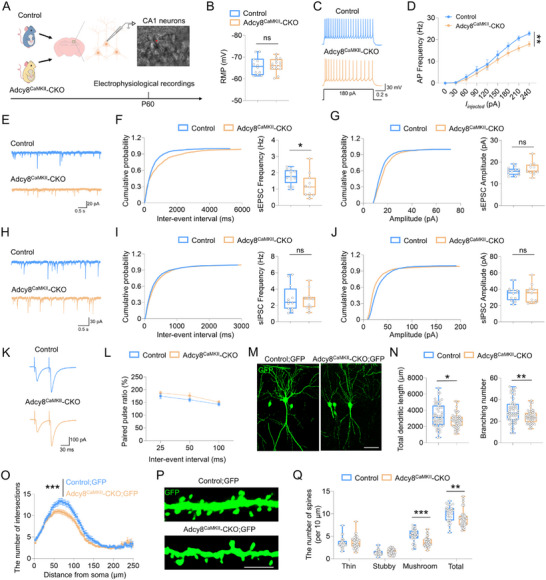
Impaired electrophysiological characteristics and morphology of dCA1 neurons in Adcy8^CaMKII^‐CKO mice. A) Schematic diagram of experimental design for electrophysiological recordings in control and Adcy8^CaMKII^‐CKO mice. Red star indicates the recorded CA1 neurons. B) Comparable resting membrane potentials of CA1 neurons in control and Adcy8^CaMKII^‐CKO mice. *n* = 12 neurons from 4 control mice and *n* = 15 neurons from 4 Adcy8^CaMKII^‐CKO mice. Student's *t*‐test. *p* = 0.7973. C) Representative traces of spikes evoked by injecting depolarizing currents. D) Firing rate plotted against increasing injected currents. *n* = 11 neurons from 4 control mice and *n* = 13 neurons from 4 Adcy8^CaMKII^‐CKO mice. Two‐way ANOVA followed by Tukey's multiple comparisons test. Interaction: F_(8, 198)_ = 1.051, *p* = 0.399; genotype factor: F_(1, 198)_ = 12.71, *p* < 0.001; currents factor: F_(8, 198)_ = 78.44, *p* < 0.001. E) Representative traces of sEPSC in dCA1 neurons of control and Adcy8^CaMKII^‐CKO mice. F and G) Quantifications of the data in E, the frequency F) and amplitude G) of sEPSC. *n* = 13 neurons from 4 control mice and *n* = 11 neurons from 4 Adcy8^CaMKII^‐CKO mice. Student's t test for F, *p* = 0.0324; Student's *t*‐test for G, *p* = 0.2667. H) Representative traces of sIPSC in dCA1 neurons of control and Adcy8^CaMKII^‐CKO mice. I and J) Quantifications of the data in H, the frequency I) and amplitude J) of sIPSC. *n* = 12 neurons from 4 control mice and *n* = 11 neurons from 4 Adcy8^CaMKII^‐CKO mice. Student's *t*‐test for I, *p* = 0.7475; Student's *t*‐test for J, *p* = 0.9008. K) Representative traces of pair‐pulse stimulation. L) PPRs plotted against interstimulus intervals. *n* = 15 neurons from 3 control mice and *n* = 11 neurons from 3 Adcy8^CaMKII^‐CKO mice. Two‐way ANOVA followed by Tukey's multiple comparisons test. Interaction: F_(2, 57)_ = 0.1165, *p* = 0.8903; genotype factor: F_(1, 57)_ = 3.561, *p* = 0.0643; time factor: F_(2, 57)_ = 9.033, *p* < 0.001. M) Representative images of dCA1 neurons in control; GFP and Adcy8^CaMKII^‐CKO; GFP mice. Scale bars  =  20 µm. N) Quantifications of total dendritic length and branch number. *n* = 58 neurons from 3 control mice and n = 52 neurons from 3 Adcy8^CaMKII^‐CKO mice. Student's *t*‐test for total dendritic length, *p* = 0.0115; Student's *t*‐test for branch number, *p* = 0.0025. O) Sholl analysis of the dendritic complexity in M. Two‐way ANOVA followed by Tukey's multiple comparisons test. Interaction: F_(100, 6010)_ = 1.300, *p* = 0.0248; genotype factor: F_(1, 6010)_ = 62.78, *p* < 0.001; distance from soma factor: F_(100, 6010)_ = 79.82, *p* < 0.001. P) Representative images of spines in control; GFP and Adcy8^CaMKII^‐CKO; GFP mice. Scale bars  =  5 µm. Q) Quantitative analysis of different types of spines in P. *n* = 30 dendrites from 3 control and *n* = 42 dendrites from 3 Adcy8^CaMKII^‐CKO mice. Student's *t*‐test for thin spines, *p* = 0.6980; Student's *t*‐test for stubby spines, *p* = 0.4165; Mann‐Whitney *U* test for mushroom spines, *p* < 0.001; Mann‐Whitney *U* test for total spines, *p* = 0.0034. Data in D and O are presented as the mean ± SEM; data in B, F, G, I, J, L, N, and Q are presented as median with interquartile range, whiskers are the minimum and maximum. *p < 0.05, **p < 0.01, ***p < 0.001, ns = no significance.

Neuronal activity is highly associated with their electrical properties. To investigate this relationship, we performed electrophysiological recordings in dCA1 neurons with the whole‐cell patch clamp configuration (**Figure** **5**A). As shown in Figure 5B, the neurons in Adcy8^CaMKII^‐CKO mice exhibited minimal alterations in the resting membrane potential. However, when subjected to depolarizing current injections, the neuronal firing rates in Adcy8^CaMKII^‐CKO mice were significantly decreased as compared with those in control mice (Figure 5C,D), suggesting a potential reduction in intrinsic excitability. Further analysis of synaptic function showed that the frequency of spontaneous excitatory postsynaptic currents (sEPSCs) was substantially decreased in Adcy8^CaMKII^‐CKO mice, without any changes in their amplitude, as compared to control mice (Figure 5E–G). In contrast, neither the frequency nor the amplitude of spontaneous inhibitory postsynaptic currents (sIPSCs) differed significantly between control and Adcy8^CaMKII^‐CKO mice (Figure 5H–J). These findings thus indicate that depletion of *Adcy8* specifically impairs glutamatergic neurotransmission while exerting little effect on GABAergic neurotransmission. Given the observed decrease in sEPSCs frequency, we next sought to determine whether there was a deficit in the probability of presynaptic glutamate release in Adcy8^CaMKII^‐CKO mice. To test this hypothesis, we examined the paired pulse ratio (PPR) of evoked EPSCs in dCA1 neurons. Unexpectedly, the PPR values in Adcy8^CaMKII^‐CKO mice were comparable to those in control mice (Figure 5K,L), excluding the possibility of a presynaptic release defect in these mice.

Neuronal function also links to its morphological features. To determine whether the morphology of dCA1 neurons was affected by *Adcy8* CKO, we generated Control; GFP and Adcy8^CaMKII^‐CKO; GFP mice by crossing Adcy8^f/f^; Thy1‐GFP mice with Adcy8^CaMKII^‐CKO mice (Figure S9A, Supporting Information). In these mice, GFP signaling driven by Thy1 promotor randomly marked excitatory neurons in multiple brain regions, including hippocampus, cortex and amygdala (Figure S9B, Supporting Information). Notably, *Adcy8* CKO largely impaired the morphology of dCA1 neurons, as both total dendritic length and branch number were significantly decreased in Adcy8^CaMKII^‐CKO; GFP mice as compared with those in control; GFP mice (Figure 5M,N). In line with this observation, Sholl analysis exhibited a decreased dendritic complexity of dCA1 neurons by *Adcy8* CKO (Figure 5O). Furthermore, the number of spines, especially mushroom‐like mature spines known as postsynaptic site, was substantially decreased in Adcy8^CaMKII^‐CKO; GFP mice (Figure 5P,Q). Collectively, these results suggest that depletion of *Adcy8* in dCA1 neurons leads to impairments in neuronal excitability, glutamatergic neurotransmission and neuronal morphology, thereby providing novel insights into the role of *Adcy8* in regulating neuronal functions.

### 2.5 Decreased *Pth2r* Expression in the Adcy8^CaMKII^‐CKO Hippocampus Mediated by Suppression of MAPK Signaling

**Figure 6:**
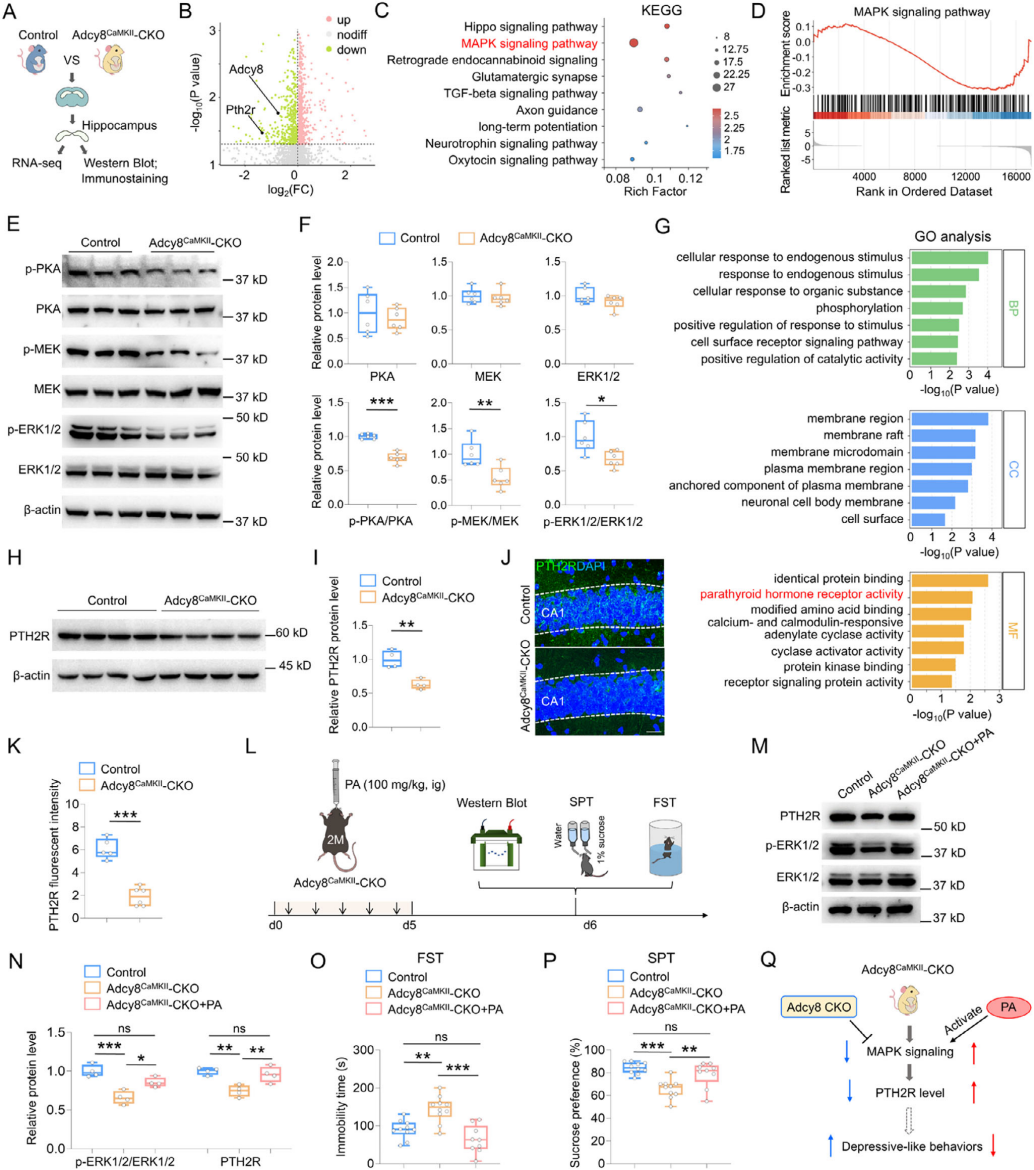
Decreased PTH2R level through inhibiting the MAPK signaling in Adcy8^CaMKII^‐CKO mice. A) Schematic diagram of experimental design for RNA‐seq, western blot and immunostaining in control and Adcy8^CaMKII^‐CKO mice. B) Volcano plots of differentially expressed genes in the Adcy8^CaMKII^‐CKO hippocampus as compared to control hippocampus. C) KEGG analysis of differentially expressed genes in the Adcy8^CaMKII^‐CKO hippocampus as compared to control hippocampus. D) GSEA analysis of MAPK signaling pathway. E) Western blot analyses of hippocampal lysates in control and Adcy8^CaMKII^‐CKO mice. F) Quantification of the data in E, the relative band intensity of proteins. *n* = 6 mice for each group. Student's *t*‐test. PKA, *p* = 0.5524; MEK, *p* = 0.6512; ERK1/2, *p* = 0.1761; p‐PKA/PKA, *p* < 0.001; p‐MEK/MEK, *p* = 0.0077; p‐ERK1/2/ERK1/2, *p* = 0.0126. (G) GO enrichment analysis of downregulated genes in the Adcy8^CaMKII^‐CKO hippocampus as compared to control hippocampus. H) Western blot analysis of PTH2R level in control and Adcy8^CaMKII^‐CKO hippocampus. I) Quantification of the data in H, the relative band intensity of hippocampal PTH2R. *n* = 4 mice for each group. Student's *t*‐test, p = 0.0024. J) Immunostaining of PTH2R (green) in the hippocampal CA1 region. DAPI (blue) was stained for cell nucleus. Scale bar = 20 µm. K) Quantification of the data in J), the fluorescent intensity of PTH2R in control (*n* = 5) and Adcy8^CaMKII^‐CKO (*n* = 6) mice. Student's *t*‐test. *p* < 0.001. L) Schematic diagram of experimental design for PA injection and western blot analysis in control, Adcy8^CaMKII^‐CKO and Adcy8^CaMKII^‐CKO+PA mice. M) Western blot analyses of PTH2R, ERK1/2 and p‐ERK1/2 in the hippocampus of control, Adcy8^CaMKII^‐CKO and Adcy8^CaMKII^‐CKO+PA mice. N) Quantification of the data in M, the relative band intensity of each protein. *n* = 4 mice for each group. One‐way ANOVA followed by Tukey's multiple comparisons test. For p‐ERK1/2/ERK1/2: F_(2, 9)_ = 20.46, *p* < 0.001. Control versus Adcy8^CaMKII^‐CKO, *p* < 0.001; Adcy8^CaMKII^‐CKO versus Adcy8^CaMKII^‐CKO+PA, *p* = 0.0131; Control versus Adcy8^CaMKII^‐CKO+PA, *p* = 0.0564; For PTH2R: F_(2, 9)_ = 12.33, *p* = 0.0026. Control versus Adcy8^CaMKII^‐CKO, *p* = 0.0032; Adcy8^CaMKII^‐CKO versus Adcy8^CaMKII^‐CKO+PA, *p* = 0.0096; Control versus Adcy8^CaMKII^‐CKO+PA, *p* = 0.7296. O) Quantifications of immobility time in the FST of control (*n* = 10), Adcy8^CaMKII^‐CKO (*n* = 10), and Adcy8^CaMKII^‐CKO+PA (*n* = 9) mice. One‐way ANOVA followed by Tukey's multiple comparisons test. F_(2, 26)_ = 15.32, *p* < 0.001. Control versus Adcy8^CaMKII^‐CKO, *p* = 0.0028; Adcy8^CaMKII^‐CKO versus Adcy8^CaMKII^‐CKO+PA, *p* < 0.001; Control versus Adcy8^CaMKII^‐CKO+PA, *p* = 0.1945. P) Quantifications of sucrose preference in the SPT of control (*n* = 10), Adcy8^CaMKII^‐CKO (*n* = 10), and Adcy8^CaMKII^‐CKO+PA (*n* = 9) mice. One‐way ANOVA followed by Tukey's multiple comparisons test. F_(2, 26)_ = 12.51, *p* < 0.001. Control versus Adcy8^CaMKII^‐CKO, *p* < 0.001; Adcy8^CaMKII^‐CKO versus Adcy8^CaMKII^‐CKO+PA, *p* = 0.008; Control versus Adcy8^CaMKII^‐CKO+PA, *p* = 0.3158. Q) A working model shows that *Adcy8* CKO downregulates MAPK signaling and PTH2R level, and induces mouse depressive‐like behaviors, whereas PA treatment reverses these phenotypes. Data in F, I, K, and N–P are presented as median with interquartile range, whiskers are the minimum and maximum. *p < 0.05, **p < 0.01, ***p < 0.001, ns = no significance.

ADCY8 is a key enzyme that catalyzes cAMP production from ATP. Consistent with this, hippocampal cAMP levels were significantly decreased in Adcy8^CaMKII^‐CKO mice compared to control mice (Figure S10A,B, Supporting Information). Interestingly, a similar reduction in cAMP levels was also detected in CRS treated mice (Figure S10C,D, Supporting Information), supporting the view that the reduced cAMP levels in CRS treated mice may be largely attributable to ADCY8 deficiency. Next, to investigate the molecular mechanisms by which *Adcy8* regulates mouse depressive‐like behaviors and synaptic functions, we performed RNA‐sequencing analysis to identify dysregulated genes in the hippocampus of Adcy8^CaMKII^‐CKO mice (**Figure** **6**A). As shown in Figure 6B, in addition to the decreased expression of *Adcy8* gene, a total of 414 genes were downregulated and 682 genes were upregulated in the Adcy8^CaMKII^‐CKO hippocampus as compared to control hippocampus. KEGG analysis of these differentially expressed genes exhibited multiple disrupted signaling pathways, including MAPK signaling pathway (Figure 6C). Gene set enrichment analysis (GSEA) suggested that the MAPK signaling pathway was downregulated in Adcy8^CaMKII^‐CKO mice (Figure 6D). This view was further supported by western blot analysis, in which the phosphorylation levels of key proteins associated with MAPK signaling pathway, such as p‐PKA, p‐MEK and p‐ERK1/2, were significantly decreased in Adcy8^CaMKII^‐CKO hippocampus as compared with those in control hippocampus (Figure 6E,F). In contrast, the total levels of these proteins remain unchanged (Figure 6E,F). Notably, all these proteins were not altered in Adcy8^PV^‐CKO hippocampus as compared with those in control hippocampus (Figure S11, Supporting Information). Thus, these findings suggest that only *Adcy8* CKO in CaMKII‐Cre^+^ neurons are responsible for the inhibition of MAPK signaling pathway.

It is well known that MAPK signaling plays a crucial role in gene transcription.^[^ ^34^ ^]^ To fully understand the downstream effects of ADCY8‐cAMP‐MAPK pathway implicated in modulating depressive‐like behaviors and neuronal activity, we conducted a comprehensive screening of dysregulated genes by Gene Ontology (GO) analysis. In the biological process (BP) category, the dysregulated genes were highly enriched in cellular response pathways. For cell component (CC), enrichments were observed in the membrane region, raft and microdomain. Additionally, dysregulated genes were also enriched in identical protein binding and parathyroid hormone receptor activity in the molecular function (MF) category (Figure 6G). Notice that parathyroid hormone signaling is critical for stress response and emotional processing,^[^ ^35^ ^]^ we screened for relevant genes and found that the expression of *Pth2r*, encoding the parathyroid hormone 2 receptor (PTH2R), was significantly decreased by *Adcy8* CKO. The reduction in PTH2R protein levels in the hippocampus of Adcy8^CaMKII^‐CKO mice was confirmed by western blot and immunostaining analyses (Figure 6H–K). Interestingly, these changes are consistent with the observations in CRS treated mice: compared to control mice, WT+CRS mice also exhibited reduced hippocampal *Pth2r* expression and downregulated MAPK signaling pathway (Figure S12, Supporting Information). Next, to determine whether PTH2R reduction was attributed to the inhibition of MAPK signaling pathway, we administered the selective ERK1/2 agonist, PA (100 mg kg^−1^, ig), to Adcy8^CaMKII^‐CKO mice (Figure 6L). As shown in Figure 6M,N, not only the expression level of p‐ERK1/2, PTH2R was also increased in Adcy8^CaMKII^‐CKO+PA hippocampus, which restored to comparable levels with those in control hippocampus. Moreover, depressive‐like behaviors of Adcy8^CaMKII^‐CKO mice were also reversed by PA injection (Figure 6O,P). Collectively, these results suggest that *Pth2r* is a key downstream effector of *Adcy8* in regulating mouse depressive‐like behaviors, largely through modulation of MAPK signaling pathway (Figure 6Q).

### 2.6 Knocking Down Hippocampal *Pth2r* Decreased Neuronal Excitability, Impaired Glutamatergic Neurotransmission and Induced Depressive‐Like Behaviors

**Figure 7:**
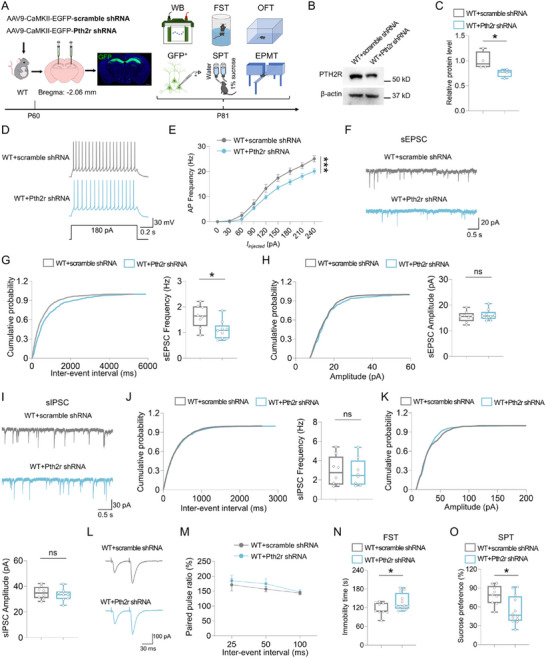
Knockdown of *Pth2r* decreased neuronal excitability, impaired glutamatergic neurotransmission, and induced depressive‐like behaviors in mice. A) Schematic diagram of experimental design for western blot, electrophysiological recording and behavioral tests in WT mice injected with AAV9‐CaMKII‐EGFP‐Pth2r shRNA or AAV9‐CaMKII‐EGFP‐scramble shRNA. B) Western blot analysis of PTH2R level in the hippocampus of WT+scramble shRNA and WT+Pth2r shRNA mice. C) Quantification of the data in B, the relative band intensity of PTH2R. *n* = 4 mice for each group. Student's *t*‐test. *p* = 0.0455. D) Representative traces of spikes evoked by injecting depolarizing currents. E) Firing rate plotted against increasing injected currents. *n* = 12 neurons from 4 WT+scramble shRNA mice and *n* = 14 neurons from 4 WT+Pth2r shRNA mice. Two‐way ANOVA followed by Tukey's multiple comparisons test. Interaction: F_(8, 216)_ = 1.790, *p* = 0.0803; genotype factor: F_(1, 216)_ = 34.55, *p* < 0.001; currents factor: F_(8, 216)_ = 167.4, *p* < 0.001. F) Representative traces of sEPSC in dCA1 neurons of WT+scramble shRNA and WT+Pth2r shRNA mice. G and H) Quantifications of the data in F, the frequency G) and amplitude (H) of sEPSC. *n* = 8 neurons from 3 WT+scramble shRNA mice and *n* = 9 neurons from 4 WT+Pth2r shRNA mice. Student's *t*‐test for G, *p* = 0.0146; Student's *t*‐test for H, *p* = 0.5069. I) Representative traces of sIPSC in dCA1 neurons of WT+scramble shRNA and WT+Pth2r shRNA mice. J and K) Quantifications of the data in I, the frequency J) and amplitude K) of sIPSC. *n* = 8 neurons from 3 WT+scramble shRNA mice and *n* = 8 neurons from 4 WT+Pth2r shRNA mice. Student's *t*‐test for J, *p* = 0.8245; Student's *t*‐test for K, *p* = 0.5010. L) Representative traces of pair‐pulse stimulation. M) PPRs plotted against interstimulus intervals. *n* = 8 neurons from 3 WT+scramble shRNA mice and *n* = 8 neurons from 4 WT+Pth2r shRNA mice. Two‐way ANOVA followed by Tukey's multiple comparisons test. Interaction: F_(2, 42)_ = 0.1455, *p* = 0.8650; virus factor: F_(1, 42)_ = 1.003, *p* = 0.3222; time factor: F_(2, 42)_ = 2.743, *p* = 0.0759. N) Quantifications of immobility time in the FST of WT+scramble shRNA mice (*n* = 9) and WT+Pth2r shRNA (*n* = 12) mice. Student's *t*‐test. *p* = 0.0495. O) Quantifications of sucrose preference in the SPT of WT+scramble shRNA mice (*n* = 9) and WT+Pth2r shRNA (*n* = 12) mice. Student's *t*‐test. p = 0.0135. Data in E and M are presented as the mean ± SEM; data in C, G, H, J, K, N, and O are presented as median with interquartile range, whiskers are the minimum and maximum. *p < 0.05, ***p < 0.001, ns = no significance.

To determine whether PTH2R participates in regulating the electrical characteristics of dCA1 neurons and mouse depressive‐like behaviors, we generated AAVs expressing *Pth2r*‐shRNA under the control of CaMKII promoter, and bilaterally injected them into the hippocampal dCA1 regions of WT mice. Three weeks later, western blot analysis was used to examine PTH2R level, electrophysiological recordings were conducted to evaluate GFP^+^ neuronal excitability and synaptic transmissions, and behavioral tests were performed to assess depressive‐like behaviors (**Figure** **7**A). As expected, a significant decrease in PTH2R protein levels was found in WT+*Pth2r* shRNA mice as compared to WT+scramble shRNA mice (Figure 7B,C). Notably, the firing rates of hippocampal dCA1 neurons were much lower in WT+*Pth2r* shRNA mice than those in WT+scramble shRNA mice (Figure 7D,E), indicating a decreased excitability of dCA1 neurons by knocking down *Pth2r*. Additionally, the frequency, but not amplitude, of sEPSCs was significantly decreased in the dCA1 neurons of WT+*Pth2r* shRNA mice as compared to WT+scramble shRNA mice (Figure 7F–H). In contrast, knocking down *Pth2r* exhibited little effect on sIPSCs (Figure 7I–K), and did not change PPR of evoked EPSCs (Figure 7L,M). In the behavioral tests, knockdown of *Pth2r* led to remarkable depressive‐like behaviors, which were supported by the observations that WT+*Pth2r* shRNA mice exhibited an increased duration of immobility time in the FST and decreased sucrose preference ratio in the SPT (Figure 7N,O). However, little to no differences in the OFT and EPMT were detected between WT+scramble shRNA and WT+*Pth2r* shRNA mice (Figure S13, Supporting Information). Together, these results demonstrated that knocking down *Pth2r* in dCA1 neurons not only decreased neuronal excitability and impaired glutamatergic neurotransmission, but also induced depressive‐like behaviors in mice, thus resembling the phenotypes in Adcy8^CaMKII^‐CKO mice.

### 2.7 Requirement of PTH2R for Increasing Neuronal Excitability and Glutamatergic Neurotransmission and Alleviating Depressive‐Like Behaviors in Adcy8^CaMKII^‐CKO Mice

**Figure 8:**
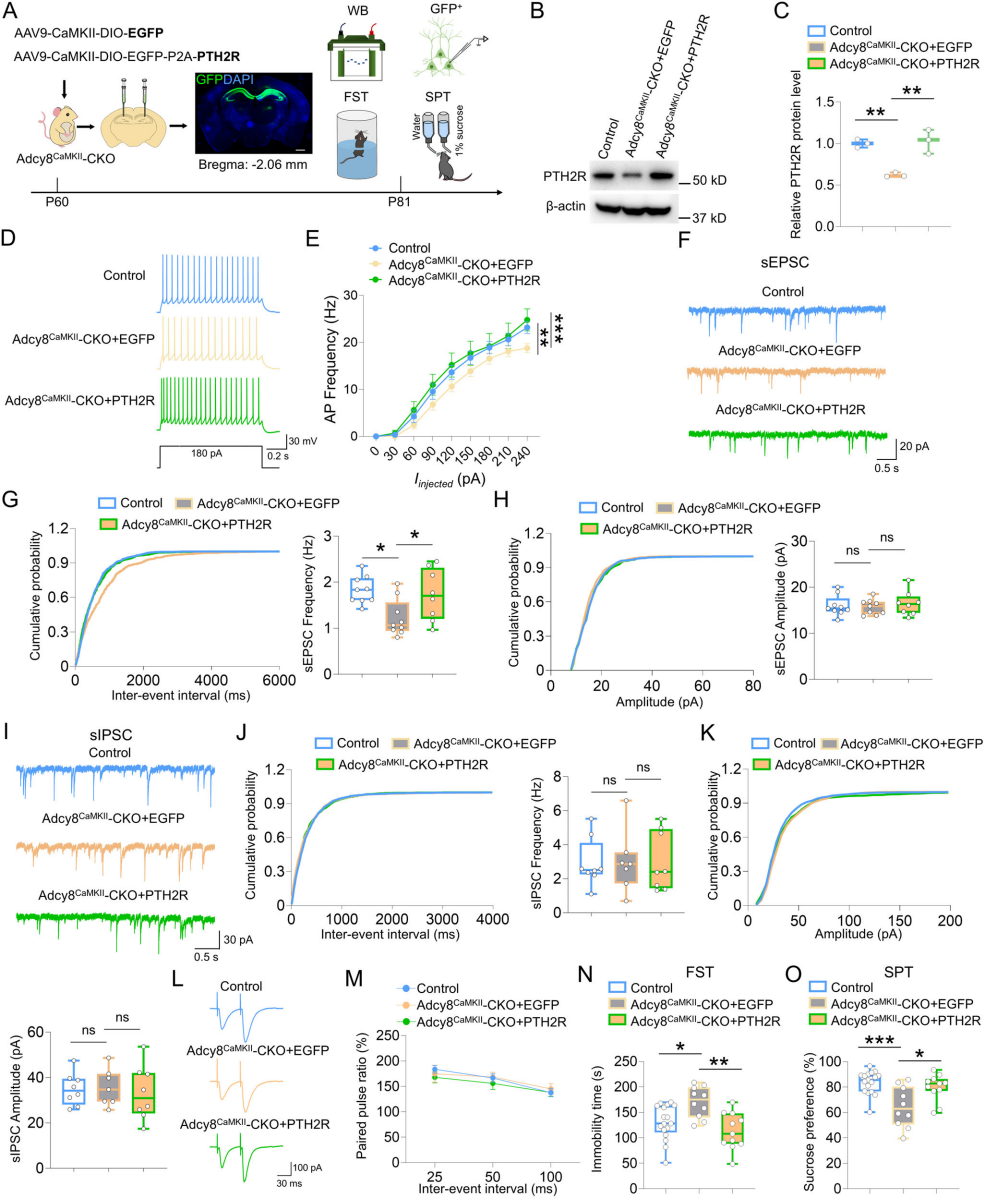
Overexpression of PTH2R rescued the deficits in neuronal excitability and glutamatergic neurotransmission, and alleviated depressive‐like behaviors of Adcy8^CaMKII^‐CKO mice. A) Schematic diagram of experimental design for western blot, electrophysiological recording and behavioral tests in Adcy8^CaMKII^‐CKO mice injected with AAV9‐CaMKII‐DIO‐EGFP‐P2A‐PTH2R or AAV9‐CaMKII‐DIO‐EGFP. Adcy8^f/f^ mice are control. B) Western blot analysis of PTH2R level in the hippocampus of control, Adcy8^CaMKII^‐CKO+EGFP and Adcy8^CaMKII^‐CKO+PTH2R mice. C) Quantification of the data in B, the relative band intensity of PTH2R. *n* = 3 mice for each group. One‐way ANOVA followed by Tukey's multiple comparisons test. F_(2, 6)_ = 19.51, *p* = 0.0024. control versus Adcy8^CaMKII^‐CKO+EGFP, *p* = 0.0048; Adcy8^CaMKII^‐CKO+EGFP versus Adcy8^CaMKII^‐CKO+PTH2R, *p* = 0.0033; control versus Adcy8^CaMKII^‐CKO+PTH2R, p = 0.9178. D) Representative traces of spikes evoked by injecting depolarizing currents. E) Firing rate plotted against increasing injected currents. *n* = 11 neurons from 3 control mice, *n* = 9 neurons from 3 Adcy8^CaMKII^‐CKO+EGFP mice and *n* = 10 neurons from 4 Adcy8^CaMKII^‐CKO+PTH2R mice. Two‐way ANOVA followed by Tukey's multiple comparisons test. Interaction: F_(16, 243)_ = 0.3678, *p* = 0.9883; genotype factor: F_(2, 243)_ = 9.132, *p* < 0.001; currents factor: F_(8, 243)_ = 88.05, *p* < 0.001. Control versus Adcy8^CaMKII^‐CKO+EGFP, *p* = 0.0085; Adcy8^CaMKII^‐CKO+EGFP versus Adcy8^CaMKII^‐CKO+PTH2R, *p* < 0.001; control versus Adcy8^CaMKII^‐CKO+PTH2R, *p* = 0.5965. F) Representative traces of sEPSC in dCA1 neurons of control, Adcy8^CaMKII^‐CKO+EGFP and Adcy8^CaMKII^‐CKO+PTH2R mice. G and H) Quantifications of the data in F, the frequency G) and amplitude H) of sEPSC. *n* = 9 neurons from 4 control mice, *n* = 9 neurons from 4 Adcy8^CaMKII^‐CKO+EGFP mice, *n* = 8 neurons from 4 Adcy8^CaMKII^‐CKO+PTH2R mice. One‐way ANOVA followed by Tukey's multiple comparisons test. For frequency: F_(2, 23)_ = 5.201, *p* = 0.0137. Control versus Adcy8^CaMKII^‐CKO+EGFP, *p* = 0.012; Adcy8^CaMKII^‐CKO+EGFP versus Adcy8^CaMKII^‐CKO+PTH2R, *p* = 0.024. For amplitude: F_(2, 23)_ = 0.2819, *p* = 0.7570. control versus Adcy8^CaMKII^‐CKO+EGFP, *p* = 0.7844; Adcy8^CaMKII^‐CKO+EGFP versus Adcy8^CaMKII^‐CKO+PTH2R, *p* = 0.7128. I) Representative traces of sIPSC in dCA1 neurons of control, Adcy8^CaMKII^‐CKO+EGFP and Adcy8^CaMKII^‐CKO+PTH2R mice. J and K) Quantifications of the data in I, the frequency J) and amplitude K) of sIPSC. *n* = 8 neurons from 4 control mice, *n* = 7 neurons from 3 Adcy8^CaMKII^‐CKO+EGFP mice and *n* = 8 neurons from 4 Adcy8^CaMKII^‐CKO+PTH2R mice. One‐way ANOVA followed by Tukey's multiple comparisons test. For frequency: F_(2, 20)_ = 0.0088, *p* = 0.9912. Control versus Adcy8^CaMKII^‐CKO+EGFP, *p* = 0.9966; Adcy8^CaMKII^‐CKO+EGFP versus Adcy8^CaMKII^‐CKO+PTH2R, *p* = 0.9966. For amplitude: F_(2, 20)_ = 0.1280, *p* = 0.8806. Control versus Adcy8^CaMKII^‐CKO+EGFP, *p* = 0.8669; Adcy8^CaMKII^‐CKO+EGFP versus Adcy8^CaMKII^‐CKO+PTH2R, *p* = 0.8608. L) Representative traces of pair‐pulse stimulation. M) PPRs plotted against interstimulus intervals. *n* = 10 neurons from 4 control mice, *n* = 9 neurons from 4 Adcy8^CaMKII^‐CKO+EGFP mice and *n* = 10 neurons from 4 Adcy8^CaMKII^‐CKO+PTH2R mice. Two‐way ANOVA followed by Tukey's multiple comparisons test. Interaction: F_(4, 78)_ = 0.2369, *p* = 0.9167; virus factor: F_(2, 78)_ = 0.7495, *p* = 0.4760; time factor: F_(2, 78)_ = 9.099, *p* < 0.001. Control versus Adcy8^CaMKII^‐CKO+EGFP, *p* = 0.9996; Adcy8^CaMKII^‐CKO+EGFP versus Adcy8^CaMKII^‐CKO+PTH2R, *p* = 0.5446. N) Quantifications of immobility time in the FST of control (n = 17), Adcy8^CaMKII^‐CKO+EGFP (n = 10) and Adcy8^CaMKII^‐CKO+PTH2R (*n* = 11) mice. One‐way ANOVA followed by Tukey's multiple comparisons test. F_(2, 35)_ = 7.983, *p* = 0.0014. Control versus Adcy8^CaMKII^‐CKO+EGFP, *p* = 0.0144; Adcy8^CaMKII^‐CKO+EGFP versus Adcy8^CaMKII^‐CKO+PTH2R, *p* = 0.0012. O) Quantifications of sucrose preference in the SPT of control (*n* = 17), Adcy8^CaMKII^‐CKO+EGFP (*n* = 10) and Adcy8^CaMKII^‐CKO+PTH2R (*n* = 11) mice. One‐way ANOVA followed by Tukey's multiple comparisons test. F_(2, 35)_ = 9.123, *p* < 0.001. Control versus Adcy8^CaMKII^‐CKO+EGFP, *p* < 0.001; Adcy8^CaMKII^‐CKO+EGFP versus Adcy8^CaMKII^‐CKO+PTH2R, *p* = 0.0119. Data in E and M are presented as the mean ± SEM; data in C, G, H, J, K, N, and O are presented as median with interquartile range, whiskers are the minimum and maximum. *p < 0.05, **p < 0.01, ***p < 0.001, ns = no significance.

Given the key role of TIP39‐PTH2R signaling in emotional processing,^[^ ^35^ ^]^ our findings revealed significant decreases in both the mRNA and protein levels of PTH2R in the hippocampus of Adcy8^CaMKII^‐CKO mice. These observations led us to speculate that depletion of *Adcy8* gene exacerbates depressive‐like behaviors by suppressing TIP39‐PTH2R signaling pathway. To test this hypothesis, Cre‐dependent AAVs expressing GFP or PTH2R were injected bilaterally into the dCA1 regions of Adcy8^CaMKII^‐CKO mice, a representative image of GFP signal presenting the appropriate injection sites were shown in **Figure** **8**A. Three weeks later, western blot analysis, electrophysiological recordings and behavioral tests were used to assess PTH2R protein level, electrical properties and depressive‐like behaviors, respectively (Figure 8A). As expected, the hippocampal PTH2R level was increased in Adcy8^CaMKII^‐CKO+PTH2R mice while no such increase was observed in Adcy8^CaMKII^‐CKO+EGFP mice (Figure 8B,C). Interestingly, the decreased excitability of dCA1 neurons in Adcy8^CaMKII^‐CKO mice was restored to control level by overexpressing PTH2R (Figure 8D,E). Moreover, increasing *Pth2r* expression also rescued the sEPSC impairments, with minimal effect on sIPSC and PPR, in Adcy8^CaMKII^‐CKO mice (Figure 8F–M). In the behavioral tests, FST and SPT showed depressive‐like behaviors in Adcy8^CaMKII^‐CKO+EGFP mice as compared to control mice, whereas overexpression of *Pth2r* in the dCA1 neurons effectively alleviated the depressive‐like phenotypes in Adcy8^CaMKII^‐CKO mice (Figure 8N,O).

Next, to further determine whether the depressive‐like behaviors of Adcy8^CaMKII^‐CKO mice could be rescued by increasing TIP39 level, the endogenous ligand for PTH2R, we performed a chronic bilateral infusion of TIP39 (100 pM, 1 µL) into the dorsal hippocampus of Adcy8^CaMKII^‐CKO mice daily for 5 days. A representative illustration of the accurate infusion sites was presented in Figure S14A (Supporting Information). Interestingly, both FST and SPT showed pronounced antidepressant effects of TIP39 in Adcy8^CaMKII^‐CKO mice as compared with vehicle‐injected Adcy8^CaMKII^‐CKO mice (Figure S14B,C, Supporting Information). Taken together, these results demonstrate that hippocampal *Adcy8* regulates neuronal excitability, glutamatergic neurotransmission and depressive‐like behaviors by modulating TIP39‐PTH2R signaling.

## 3 Discussion

**Figure 9:**
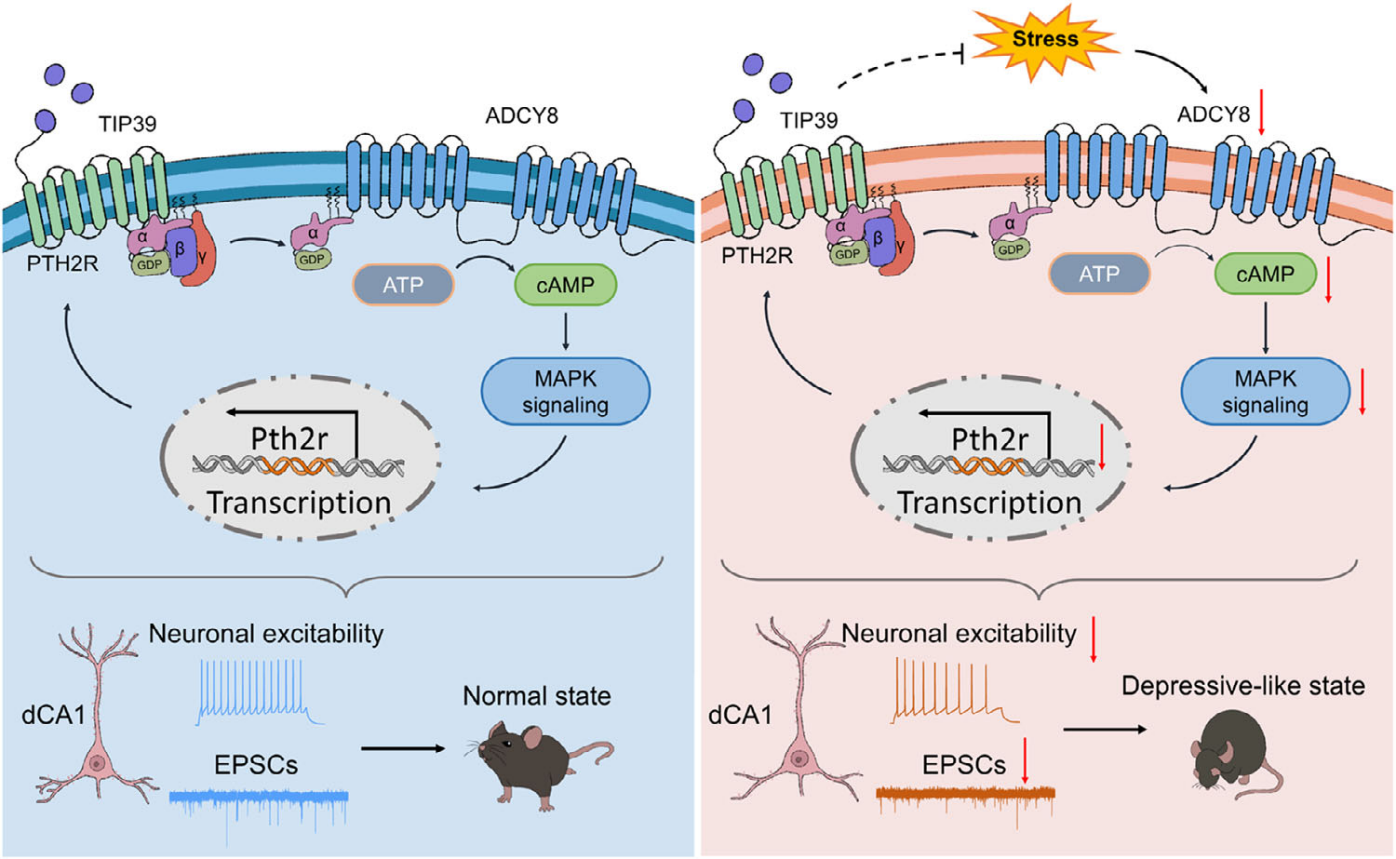
A working model and summary of results.

Hippocampus is a critical regulator for both cognition and emotional processing. Deficits in hippocampal neurons contribute to the development of many neurological disorders, including depression. However, the detailed mechanisms by which hippocampal neurons regulate depression remain elusive. Here, as depicted in the working model in **Figure** **9**, we provide evidence that *Adcy8* in dCA1 neurons controls their activities and functions, including intracellular calcium signal, intrinsic excitability and glutamatergic neurotransmission, to prevent mouse depressive‐like behaviors. In contrast, chronic stress selectively decreases *Adcy8* expression in dCA1 neurons. This reduction further downregulates PTH2R through inhibiting the MAPK signaling pathway. Deficiency in either *Adcy8* or *Pth2r* induces a remarkable decrease in neuronal excitability and glutamatergic neurotransmission, and eventually promotes depression pathogenesis.

ADCYs are the key enzymes for generating cAMP. Accumulating evidence has demonstrated an impaired ADCY‐cAMP pathway in depressed patients and animal models.^[^ ^23^, ^24^ ^]^ The activation of ADCY was considered as a biomarker for the clinic response to antidepressants.^[^ ^21^ ^]^ While GWAS suggests several *ADCY* genes implicated in depression,^[^ ^36^, ^37^, ^38^, ^39^ ^]^ the underlying mechanisms are still unclear. Using a combination of CRS and CSDS mouse models, RNA‐sequencing and molecular techniques, we demonstrated that *Adcy8*, but not other *Adcy* genes, was largely decreased in the hippocampus of mice exposed to chronic stress (Figure 1; Figure S4, Supporting Information). Given the distribution of *Adcy8* mRNAs in the olfactory bulb, cerebellum, thalamus, hypothalamus, habenula and cortex,^[^ ^40^ ^]^ our findings revealed that CRS selectively decreased *Adcy8* expression in the hippocampus, with no such decrease in the cortex, thalamus and hypothalamus (Figure 1H). Further RNA‐scope analysis also confirmed the reduction of *Adcy8* mRNAs in dCA1 neurons, but not in other hippocampal subregions, of chronic stress treated mice (Figure 1I,J; Figures S3 and S4, Supporting Information), supporting a specific role of dCA1's *Adcy8* in regulating stress‐associated neurological disorders. Indeed, ADCY8 has been reported to be associated with bipolar disorder, autism spectrum disorder and post‐traumatic stress disorder in humans.^[^ ^37^, ^41^, ^42^ ^]^ Our results indicated that whole‐body KO or CKO of *Adcy8* induced pronounced depressive‐like behaviors in mice, thereby providing new insights into the roles of *Adcy8* in depression. Notably, anxiety‐like behaviors were observed in *Adcy8* KO mice but absent in Adcy8^CaMKII^‐CKO mice. This discrepancy may be explained by the different *Adcy8* knockout strategies: whole‐body KO results in gene depletion across all cells starting from the embryonic stage, whereas CaMKII promoter‐driven CKO (CaMKII‐CKO) mediates gene deletion exclusively in forebrain excitatory neurons from postnatal day 19.^[^ ^43^ ^]^ It is thus speculated that *Adcy8* expression in other organs, or during the embryonic and early postnatal stages, plays a critical role in regulating anxiety‐like behaviors.

It is well recognized that hippocampus, along its dorsal‐ventral axis, plays distinct functions. The dorsal portion is closely associated with cognitive functions while the ventral portion is primarily responsible for emotional regulation.^[^ ^44^ ^]^ However, this view is becoming less absolute, as a growing body of evidence indicates that dorsal hippocampus, including dCA1 and dDG neurons, also modulates emotional or stress‐related behaviors in mice.^[^ ^45^, ^46^, ^47^, ^48^ ^]^ How does hippocampal *Adcy8* regulate the onset of depression? To address this question, it is important to precisely identify the specific cell types in which *Adcy8* is expressed. By snRNA‐seq analysis and co‐detection of RNA‐scope and immunostaining in the hippocampus, we found abundant *Adcy8* mRNAs in both excitatory and inhibitory neurons (Figure 2). However, CRS specifically decreased *Adcy8* mRNAs in CaMKII^+^ dCA1 neurons, rather than in PV^+^ inhibitory neurons (Figure S5, Supporting Information). Consistent with this, in contrast to *Adcy8* CKO in PV^+^ inhibitory neurons, depletion of *Adcy8* in CaMKII^+^ excitatory neurons led to remarkable depressive‐like behaviors (Figure 3E–L; Figure S7A–D, Supporting Information). Numerous studies have indicated that dysfunctions in hippocampal neurons are closely associated with depression. Functional magnetic resonance imaging (fMRI) of patients with depression showed that the activity of hippocampus is largely inhibited.^[^ ^49^, ^50^ ^]^ In animal models, chronic stress significantly decreased the firing rates of hippocampal excitatory neurons,^[^ ^51^ ^]^ as well as reduced activity‐dependent factors, such as brain‐derived neurotrophic factor (BDNF),^[^ ^26^ ^]^ which contribute to the development of depression. *Adcy8* is known for its roles in neuronal differentiation, development and synaptic plasticity.^[^ ^52^, ^53^, ^54^ ^]^ It remains to be determined whether *Adcy8* regulates neuronal activity, especially in excitatory neurons. Our findings revealed that *Adcy8* CKO in CaMKII^+^ excitatory neurons not only reduced the calcium signals responding to stress (Figure 4D–M), but also substantially decreased the intrinsic excitability and sEPSC frequency in dCA1 neurons (Figure 5C–F), in support of the view that the decrease in hippocampal activity caused by *Adcy8* CKO resulted in depression. However, there is also a controversial report suggesting that hippocampal overactivity correlates with depression severity.^[^ ^55^ ^]^ These distinct phenotypes may be attributed to specific neuron subtypes, circuits or hippocampal subregions (e.g., dorsal versus ventral). Thus, it would be interesting to further investigate more detailed mechanisms underlying the relationship between hippocampal activity and depression.

Numerous studies have revealed the inhibition of MAPK signaling in the brains of depressed patients and animal models.^[^ ^16^, ^56^, ^57^ ^]^ MAPK signaling is well known for its roles in regulating neuronal activity. Of note is that the impact of MAPK signaling on neuronal activity depends on brain regions or neuron subtypes. Loss of MAPK signaling is reported to reduce neuronal excitability in the striatum while increasing neuronal excitability in the developing neocortex.^[^ ^58^, ^59^ ^]^ Interestingly, our findings showed a significant downregulation of MAPK signaling in the hippocampus of Adcy8^CaMKII^‐CKO mice (Figure 6A–F), providing a potential link to the decreased excitability in *Adcy8* depleted dCA1 neurons. What is the underlying downstream mechanism explaining these phenotypes in Adcy8^CaMKII^‐CKO mice? Given that MAPK signaling is closely associated with gene expression, it not only modulates the activity of transcription factors and co‐regulatory proteins, but also targets chromatin‐associated proteins and alters nucleosome structure through inducing histone modifications, which drive either transcriptional activation or repression.^[^ ^34^, ^60^ ^]^ In this study, using RNA‐sequencing and biochemical approaches, we screened out PTH2R mediating the effects of ADCY8 deficiency on neuronal functions and mouse depressive‐like behaviors. The reasons are as follows: first, both *Pth2r* mRNAs and PTH2R protein levels are substantially decreased in Adcy8^CaMKII^‐CKO hippocampus due to the inhibition of MAPK signaling pathway, whereas administration of an ERK1/2 agonist (PA) restored PTH2R level in the Adcy8^CaMKII^‐CKO hippocampus (Figure 6B,H–N), as well as reversed the depressive‐like behaviors in Adcy8^CaMKII^‐CKO mice (Figure 6O,P). However, the detailed mechanism by which MAPK signaling transcriptionally regulates *Pth2r* expression remains unclear and requires further investigation. Second, knockdown of hippocampal *Pth2r* decreased the excitability and glutamatergic neurotransmission in dCA1 neurons and induced depressive‐like behaviors, resembling the phenotypes in Adcy8^CaMKII^‐CKO mice (Figure 7). Third, overexpression of *Pth2r* in Adcy8^CaMKII^‐CKO hippocampus rescued the deficits in neuronal excitability and glutamatergic neurotransmission, and reversed the depressive‐like behaviors (Figure 8). Fourth, increasing TIP39 levels, the natural ligand for PTH2R, also alleviated the depressive‐like behaviors in Adcy8^CaMKII^‐CKO mice (Figure S14, Supporting Information).

Notably, female mice with *Pth2r* KO have been reported to display depressive‐like behaviors during lactation.^[^ ^61^ ^]^ Disruption of TIP39‐PTH2R signaling, such as depleting TIP39, also induced depressive‐ and anxiety‐like behaviors following conditional fear stimuli.^[^ ^62^ ^]^ However, the exact mechanism by which PTH2R regulates depressive‐like behaviors remains elusive. Our results suggest that knockdown of hippocampal *Pth2r* resulted in depressive‐like behaviors through decreasing the excitability and glutamatergic neurotransmission in dCA1 neurons (Figure 7). How does *Pth2r* modulate these electrical properties? Indeed, PTH2R belongs to GPCR family, whose functions are closely associated with neuronal excitability, synaptic transmission, and ion channel regulation.^[^ ^63^, ^64^, ^65^ ^]^ Previous studies have shown that TIP39 increases intracellular calcium concentration in a dose‐dependent manner, whereas *Pth2r* knockdown inhibits this upregulation of calcium in response to TIP39, suggesting a crucial role of *Pth2r* in maintaining calcium homeostasis.^[^ ^66^ ^]^ Additionally, dysregulation of ion channels (e.g., sodium, potassium, calcium, and chloride channels) typically alters the neuronal excitability;^[^ ^67^, ^68^, ^69^, ^70^ ^]^ Further studies are therefore needed to investigate whether *Pth2r* regulates neuronal excitability by modulating ion channel activity, and if so, to clarify the detailed mechanisms. Of note, class B GPCR is commonly referred to as the secretin‐like family of GPCRs, these receptors are involved in signal transduction by coupling to heterotrimeric G proteins that primarily activate ADCY.^[^ ^71^ ^]^ Thus, determining whether TIP39‐PTH2R signaling forms a positive feedback loop regulating ADCY8 activity in future studies would be of great interest. Such results may provide additional evidence to support the observations that increasing hippocampal TIP39 levels alleviated depressive‐like behaviors in Adcy8^CaMKII^‐CKO mice.

In summary, our findings indicate that ADCY8‐deficiency in dCA1 neurons is a risk factor for the development of depression. Depletion of *Adcy8* in dCA1 neurons decreased neuronal excitability, impaired glutamatergic neurotransmission, and induced depressive‐like behaviors, likely through the inhibition of MAPK‐PTH2R signaling.

## 4 Experimental Section

### Animals and Mouse Breeding

C57BL/6J mice were purchased from Charles River. Adcy8 KO mice (S‐KO‐00920), Adcy8^f/f^ mice (S‐CKO‐01055), CaMKII‐Cre mice (C001015) were purchased from Cyagen Biosciences. PV‐Cre mice were kindly provided by Dr. Zhi‐Heng Xu (Institute of Genetics and Developmental Biology, Chinese Academy of Sciences). Thy1‐GFP mice were kindly provided by Dr. Wei‐Xiang Guo (Institute of Genetics and Developmental Biology, Chinese Academy of Sciences). Adcy8^CaMKII^‐CKO and Adcy8^PV^‐CKO mice were generated by crossing Adcy8^f/f^ mice with CaMKII‐Cre mice and PV‐Cre mice, respectively. All the mouse lines were maintained in C57BL/6J strain background for >6 generations, and confirmed by genotyping analysis with PCR. Mice were maintained on a 12 h light‐dark cycle with ad libitum access to water and food. All the experiments with animals were performed with the approval (No. YNPZXM2024034) of the Institutional Animal Care and Use Committee of Jilin University.

### Chronic Restraint Stress

Young adult WT mice were placed in a 50 mL plastic air accessible tube for 6 h (10:00–16:00) per day for 10 consecutive days. In the tube, the mice could not move freely and without food and water. The non‐stressed control mice were littermates and were able to move freely in their home cages. Behavioral tests and biochemical analysis were performed at indicated time.

### Chronic Social Defeat Stress

Adult male CD‐1 mice (4–6 months old) were used as aggressors. Before each defeat, aggressors were screened for aggressive behavior for 3 consecutive days. Two days before the start of defeat, the CD‐1 mice were housed on one side of a perforated Plexiglass partition. During CSDS, young adult WT mice (7–8 weeks old) were subjected to direct physical interaction with a CD‐1 mouse for 10 min per day, and for the rest of the day were placed on the other side of the Plexiglass divider, allowing for sensory but not direct physical contact. The experimental mice (WT) were exposed to a new CD‐1 aggressor every day for 10 consecutive days.

### Behavioral Tests

All behavioral tests were performed as previously described with slight modifications.^[^ ^48^, ^72^ ^]^ Mice were transferred to the behavioral room 2 h before any test to acclimate to the environment. All behavioral instruments in EPMT, OFT, and FST were cleaned with 75% ethanol prior to each trial.

*Forced swim test (FST)*. FST was performed in a glass cylinder (12 cm diameter, 25 cm high) containing water (23–25 °C). The mice were placed in the cylinder individually and recorded for 6 min, the duration of immobility was quantified during the last 4 min. Immobility time was defined as the time that mice remained floating or motionless.

*Sucrose preference test (SPT)*. The mice were acclimated to the presence of two bottles (50 mL tubes fitted with ball‐point sipper tubes) for 48 h. One bottle was filled with 1% sucrose, and the other was filled with drinking water. Bottle positions were swapped to prevent position bias every 24 h. After another 24 h period, the amounts of sucrose and water consumed were recorded, with bottle positions swapped every 12 h. Sucrose preference (%) was calculated as sucrose consumption / (sucrose + water consumption) × 100%.

*Open field test (OFT)*. Each mouse was placed in a chamber (L × W × H = 50 × 50 × 40 cm) and movement was monitored for 10 min using an overhead camera. Light intensity was ≈200 lux. The video was analyzed by a tracking software (Smart 3.0, Panlab Harvard Apparatus). Total distance and time spent in center (25 × 25 cm) were quantified.

*Elevated plus maze test (EPMT)*. the plus maze was placed 50 cm above the ground. Each mouse was initially placed in the center square facing one of the open arms (L × W = 30 × 5 cm). Mice movement was recorded for 5 min using an overhead camera and tracking software (Smart 3.0, Panlab Harvard Apparatus). Time spent in the open arms and the number of open arm entries were quantified.

*Social interaction test (SIT)*. This assay was conducted as previously described.^[^ ^73^ ^]^ In brief, the social interaction ratio (SIR), which is defined as the ratio of a mouse's interaction time with a target mouse to its interaction time with an empty wire‐mesh enclosure, was used for classification. Mice showing an SIR >1 were considered as resilient and those with an SIR < 1 were considered as susceptible.

### snRNA‐Seq Analysis

Hippocampi of WT mice were rapidly isolated and placed on ice and frozen in liquid nitrogen. The hippocampal tissues were homogenized in ice‐cold homogenization buffer (0.25 m sucrose, 5 mm CaCl_2_, 3 mm MgAc_2_, 10 mm Tris‐HCl (pH 8.0), 0.1 mm EDTA, 1× protease inhibitor, and 1 U µL^−1^ RiboLock RNase inhibitor) with pestle strokes. Next, the homogenates were filtered through a 70 µm cell strainer to collect the nuclear fraction. The nuclear fraction was mixed with an equal volume of 50% iodixanol and added on top of a 30% iodixanol solution. This solution was then centrifuged for 20 min at 10 000 ×*g* at 4 °C. After the myelin layer was removed from the top of the gradient, the nuclei were collected from the 30% iodixanol interface. The nuclei were resuspended in nuclear wash buffer and resuspension buffer (0.04% bovine serum albumin, 0.2 U µL^−1^ RiboLock RNase inhibitor, 500 mM mannitol and 0.1 mM phenylmethanesulfonyl fluoride (as a protease inhibitor) in PBS) and pelleted for 8 min at 500 ×*g* and 4 °C. The nuclei were filtered through a 40 µm cell strainer to remove cell debris and large clumps. The nuclear concentration was manually assessed using trypan blue counterstaining and a hemocytometer. Finally, the nuclear concentration was adjusted to 700–1200 nuclei µL^−1^, and the nuclei were examined with a 10× Chromium platform. Reverse transcription, cDNA amplification and library preparation were performed based on the protocol from the manufacturer (10× Genomics, USA).

For data preprocessing, raw reads were preprocessed using Cell Ranger (version 3.1.0) with the default parameters and aligned to the pre‐mRNA reference (Ensemble_ release 100, Mus musculus). For quality control, cells with gene counts between 500 and 4000 per cell, UMI counts <8000 per cell, and a percentage of mitochondrial genes <10% were retained for downstream analysis. Then, the global‐scaling normalization method “LogNormalize” was used to normalize the gene expression measurements for each cell by total expression, multiplied this by a scale factor (10 000 by default), and log‐transformed the result with the following formula:

(1) $$A\;\mathrm{gene}\;\mathrm{expression}\;\mathrm{level}=\log\left(1+\frac{UMI_{A}}{UMI_{Total}}\times 10000\right)$$

After data integration and scaling, principal component analysis (PCA) was applied with the “RunPCA” (Seurat package) function, and appropriate principal components were selected for subsequent analysis.

For cell clustering, distances between cells were calculated based on the previously identified PCA score. Briefly, Seurat embed cells in a shared‐nearest neighbor (SNN) graph, with edges drawn between cells via similar gene expression patterns. To partition this graph into highly interconnected quasi‐cliques or communities, we first constructed the SNN graph based on the Euclidean distance in PCA space and refined the edge weights between any two cells based on the shared overlap in their local neighborhoods (Jaccard distance). Then, these datasheets were visualized by Uniform Manifold Approximation and Projection (UMAP). Marker genes were then identified using the function FindAllMarkers in Seurat. Cell type was annotated based on top ranked marker genes. Expression value of each gene in given cluster were compared against the rest of cells using Wilcoxon rank sum test. Significant upregulated genes were identified using several criteria. First, genes had to be at least 1.28‐fold overexpressed in the target cluster. Second, genes had to be expressed in more than 25% of the cells belonging to the target cluster. Third, *p* < 0.01.

### RNA‐seq

Hippocampi from each group of mice were dissected to RNA sequencing using the BGI platform. To identify differentially expressed genes and related functions, we performed volcano plot, KEGG and GO analyses by online tool (https://www.omicshare.com). *p* < 0.05 was considered statistically significant.

### Quantitative Real‐Time PCR (qPCR)

RNAeasy Animal RNA Isolation Kit (Beyotime, Cat No. R0026) was used to extract total RNA, and reverse transcription was performed following the protocol of SweScript All‐in‐One RT SuperMix for qPCR Kit (Servicebio, Cat No. G3337‐100). The reaction cycle conditions were as follows: initial denaturation at 95 °C for 30 s, followed by 40 cycles of 10 s at 95 °C and 30 s at 60 °C. The ratio of mRNA expression in hippocampal tissues was analyzed via the 2^−ΔΔCT^ method. The primers were as follows: Adcy1, 5′‐CCTTCTCCAACGTGATGACCTG‐3′ and 5′‐GCTGATGTACCTGTTGACGCGT‐3′; Adcy8, 5′‐ACGACATGACCAATGTGGAG‐3′ and 5′‐TGTCAACATCGTGCTTCGTT‐3′; Gapdh, 5′‐CATCACTGCCACCCAGAAGACTG‐3′ and 5′‐ATGCCAGTGAGCTTCCCGTTCAG‐3′.

### RNA scope

Mice were anesthetized with isoflurane and perfused with 50 mL PBS followed by 50 mL 4% paraformaldehyde (PFA). Brains were post‐fixed in 4% PFA overnight and then immersed in 30% sucrose solution for 24 h. After that, brains were embedded in optimal cutting temperature compound (Sakura) at −20 °C and cut into 12 µm slices using a freezing microtome (Leica CM1950). Subsequently, RNA scope was performed in mouse brain sections by using RNAscope Multiplex Fluorescent Detection Kit (PN323110) and mouse *Adcy8* probes (ACD 462 041).

### Fiber Photometry

Calcium signal acquisition was performed through fiber photometry system (ThinkerTech Nanjing Biotech Ltd). For neural interface establishment, a sterilized optical implant (200 µm outer diameter, 0.37 numerical aperture, 2‐mm length) was surgically positioned in the CA1 following stereotaxic coordinates (anteroposterior: −2.0 mm; mediolateral: 1.1 mm; dorsoventral: 1.15 mm). Craniofacial fixation was achieved through dental cement reinforcement with an integrated M1 anchoring screw. Light transmission between rotational commutator and cortical implant was mediated by a 220‐µm diameter optical conduit matching the implant's numerical characteristics.

Prior to experimental measurements, laser calibration procedures were implemented with 0.02 mW bleaching protocol. During signal acquisition phases, terminal output intensities were maintained at 20 µW (405 nm reference wavelength) and 40 µW (470 nm excitation wavelength) respectively. Fluorescent signals from GCaMP6m‐expressing neurons were captured through wavelength‐specific detection modules. Subsequent computational processing utilized ThinkerTech's proprietary analytical suite implemented in MATLAB R2022a computational environment. Signal normalization involved temporal baseline correction derived from habituation phase recordings (designated as F0 reference values).

Quantitative analysis of calcium transients. Signal quantification employed temporally defined analytical windows: a 2‐s pre‐event stabilization phase and an extended observation window encompassing 2 s preceding to 5 s following event initiation. Percentage signal variation:

(2) $$\Delta F/F\left(\%\right)=\left[\left(F_{t}-\langle F\rangle \mathrm{baseline}\right)/\langle F\rangle \mathrm{baseline}\right]\times 100\%$$

(3) $$Z-\mathrm{score}=\left(F_{t}-\mu \_\mathrm{baseline}\right)/\sigma \_\mathrm{baseline}$$

where ⟨F⟩baseline corresponds to the arithmetic mean of fluorescence intensities during the reference stabilization phase, while µ_baseline and σ_baseline respectively represent the population mean and standard deviation derived from the same baseline epoch.

### Electrophysiological Recording

Mice were anesthetized with isoflurane and subjected to cardiac perfusion with ice‐cold Choline Chloride‐based cutting solution containing (in mM): 120 Choline Chloride, 2.5 KCl, 7 MgCl_2_, 0.5 CaCl_2_, 1.25 NaH_2_PO_4_, 5 Sodium ascorbate, 3 Sodium pyruvate, 26 NaHCO_3_, and 25 glucose. Mice were rapidly decapitated. The brain was removed quickly and placed in cutting solution. 300‐µm slices of hippocampus using VT1200S Vibratome (Leica Microsystems, Germany) as described elsewhere.^[^ ^74^, ^75^ ^]^ Slices were transferred to a storage chamber containing cutting solution and incubated at 34 °C for 15 min, Slices were then transferred and stored in artificial cerebrospinal fluid (ACSF) (in mm): 124 NaCl, 2.5 KCl, 2 MgSO_4_, 2.5 CaCl_2_, 1.25 NaH_2_PO_4_, 26 NaHCO_3_, and 10 glucose, maintained at 25 ± 1 °C for an additional 1 hour before recording. Cutting solution and ACSF were saturated with oxygen (95% O_2_/5% CO_2_).

Slices were transferred to the recording chamber superfused (2 mL/min) with regular ACSF at 32–34 °C. Slice was visualized with a 40× water‐immersion lens (FN‐S2N, Nikon) and digital CMOS camera (C13440, Hamamatsu). The borosilicate glass electrodes were pulled by a micropipette puller (P‐1000, Sutter Instrument) with resistance in the range of 3–5 MΩ. Series resistance was below 20 MΩ and monitored throughout the experiments. Recordings were performed with MultiClamp 700B amplifier and 1550B digitizer (Molecular Devices). Data were sampled at 10 kHz and low‐pass filtered at 2 kHz. For sEPSCs recording, pyramidal neurons were held at −70 mV in the presence of 20 µm bicuculline, with the pipette solution containing (in mm): 125 K‐gluconate, 5 KCl, 10 HEPES, 0.2 EGTA, 1 MgCl_2_, 4 Mg‐ATP, 0.3 Na‐GTP and 10 phosphocreatine, at pH 7.35 adjusted with KOH, (290–295 mOsm). For sIPSCs recording, the pipette solution contained 125 mm K‐gluconate and the 5 mm KCl were replaced by 125 mm Cesium methanesulfonate and 5 mm CsCl, pH was adjusted to 7.35 with CsOH. 20 µm CNQX and 50 µm DL‐AP5 were added into bath solution to blocked AMPA and NMDA receptors. Action potential frequency was analyzed from current clamp recording and measured by injecting a series of depolarizing pulses in the presence of 20 µm CNQX, 50 µm DL‐AP5 and 20 µm bicuculline. The pipette solution contained (in mM): 125 K‐gluconate, 5 KCl, 10 HEPES, 0.2 EGTA, 1 MgCl_2_, 4 Mg‐ATP, 0.3 Na‐GTP and 10 phosphocreatine, at pH 7.35 adjusted with KOH, (290–295 mOsm). For paired‐pulse ratios recording, EPSCs were evoked by stimulating SC‐CA1 pathway at holding potential of −70 mV in the presence of 20 mm bicuculline. The interval of paired stimulations was set at 25, 50, and 100 ms, respectively. Value of ratios was defined as (p2/p1) × 100, where p1 and p2 were the amplitude of the EPSCs evoked by the first and second pulse, respectively.

### Immunostaining

Immunostaining was performed as described previously.^[^ ^72^ ^]^ In brief, mice were anesthetized with isoflurane and perfused with 50 mL PBS followed by 50 mL 4% paraformaldehyde (PFA). Brains were post‐fixed in 4% PFA overnight and cut into 40 µm slices using a vibratome (Leica VT1000 S). Brain sections were washed 3 times with PBS (5 min each) and treated with blocking buffer (10% Donkey Serum + 0.5% Triton X 100) for 1 h at room temperature, and then incubated with primary antibody overnight at 4 °C. Primary antibodies were used as follows: CaMKII (ThermoFisher, PA5‐19128, 1:200), DCX (Cell signaling, 14 802, 1:300), PV (Cell signaling, 80 561, 1:500), GFAP (Proteintech, 16825‐1‐AP, 1:1000), Iba1 (Abcam, ab5076, 1:500), c‐fos (Santa Cruz, sc‐271243, 1:400), NeuN (Cell signaling, 94 403, 1:1000). On the next day, sections were washed 3 times with PBS and incubated with appropriate fluorescence‐conjugated secondary antibodies for 2 h at room temperature. DAPI was used for nucleus staining. All images were taken using a Zeiss confocal system (LSM 900).

### Quantification of Spine Subtypes

Spines located on secondary and tertiary dendritic branches were quantified using randomly selected dendritic segments. Spine density was calculated by dividing the total number of spines by the length of dendritic branch. Spine subtypes were classified according to previously established criteria,^[^ ^76^ ^]^ with detailed definitions as follows: (a) mushroom spines: spine head diameter is ≥1.5‐fold the diameter of the spine neck (dh/dn ≥1.5); (b) stubby spines: spine head and neck widths are approximately equal, and spine length is not significantly greater than head diameter (dh/dn <1.5, L/dh <2); (c) thin spines: spine head and neck diameters are nearly equal, and spine length is greater than spine width (dh/dn <1.5, L/dh ≥2). All spine analyses were performed by an investigator who was blinded to the genotypes of the samples.

### Western Blot

Western blot was performed as described previously with slight modification.^[^ ^77^ ^]^ In brief, brain tissues were homogenized in lysis buffer. Lysates were centrifuged at 12 000 rcf, 4 °C for 15 min. Then, the supernatants were got and mixed it with loading buffer. Based on the protein molecular weight, 10% SDS‐PAGE gels were used to separate proteins and then transferred them onto Nitrocellulose (NC) Blotting Membranes. After that, NC membranes were blocked with 5% bovine serum albumin (BSA) in 1×TBST for 1 h at room temperature. Primary antibodies as follows: PKA (Cell signaling, 4782, 1:1000), p‐PKA (Cell signaling, 5661, 1:500), MEK (Cell signaling, 8727, 1:1000), p‐MEK (Cell signaling, 9154, 1:1000), ERK1/2 (Cell signaling, 4695, 1:1000) and p‐ERK1/2 (Cell signaling, 4370, 1:1000), PTH2R (Proteintech, 14166‐1‐AP, 1:1000) and β‐actin (Bioss, bsm‐33036 m, 1:500) were used to incubate NC membrane overnight at 4 °C. Subsequently, membranes were washed and incubated for 1 h at room temperature with secondary antibodies which were used for recognizing the primary antibody, and ECL (Beyotime) was used for visualizing the target proteins. For quantitative analysis, detected protein bands were analyzed using ImageJ software. Bands of interested proteins were normalized to loading control (β‐actin).

### Drug Information

Pamoic acid (PA) disodium (Cat. No. E2660) was purchased from Selleck Chemicals (https://www.selleckchem.com). Its stock concentration is 10 mg mL^−1^ in saline, and a dose of PA at 100 mg kg^−1^ body weight was administered intragastrically to activate MAPK signaling in vivo. TIP39 (Cat. No. 277302‐47‐3, amino acid sequence: Ser‐Leu‐Ala‐Leu‐Ala‐Asp‐Asp‐Ala‐Ala‐Phe‐Arg‐Glu‐Arg‐Ala‐Arg‐Leu‐Leu‐Ala‐Ala‐Leu‐Glu‐Arg‐Arg‐His‐Trp‐Leu‐Asn‐Ser‐Tyr‐Met‐His‐Lys‐Leu‐Leu‐Val‐Leu‐Asp‐Ala‐Pro) was purchased from MedChemExpress (MCE; https://www.medchemexpress.com). Its stock concentration is 1 mm in saline. For in vivo experiments, 1 µL of TIP39 solution at 100 pm was infused into the dCA1 regions of mice daily for 5 days.

### Virus Preparation and Grafting In Vivo

AAV9‐CaMKII‐EGFP (BC‐0027, ≥5E+12 vg mL^−1^), AAV9‐CaMKII‐EGFP‐P2A‐Cre (BC‐0165, ≥5E+12 vg mL^−1^) and AAV9‐CaMKII‐GCaMP6m (BC‐0082, ≥5E+12 vg mL^−1^) were purchased from BrainCase; AAV9‐EF1a‐His (≥5E+12 vg mL^−1^), AAV9‐EF1a‐Adcy8‐His (≥5E+12 vg mL^−1^), AAV9‐CaMKII‐EGFP‐scramble shRNA (Target sequence: ccctaaggttaagtcgccctcg, ≥5E+12 vg mL^−1^), AAV9‐CaMKII‐EGFP‐Pth2r shRNA (Target sequence: tcgctgtcgtgatgtttattta, ≥5E+12 vg mL^−1^), AAV9‐CaMKII‐DIO‐EGFP (≥5E+12 vg mL^−1^) and AAV9‐CaMKII‐DIO‐EGFP‐P2A‐PTH2R (≥5E+12 vg mL^−1^) were packaged by Cyagen Biosciences.

Virus injection was performed as described previously with a slight modification.^[^ ^72^ ^]^ Mice were anesthetized with isoflurane, and head was fixed in a stereotaxic device (David Kopf Instruments). After antiseptic treatment, skull was exposed and cleaned using 1% H_2_O_2_. Holes were drilled into the skull and viruses (0.5 µL) were bilaterally injected into dCA1 (caudal: −2.00 mm; lateral: + /−1.1 mm; ventral: −1.15 mm) or vCA1 (caudal: −3.28 mm; lateral: + /−3.2 mm; ventral: −2.25 mm) region. After injection, needle was left in place for 5 min to allow for diffusion of injected viruses before being slowly withdrawn. For the following 5 days, mice received Meloxicam via intraperitoneal (i.p.) injection at 5 mg kg^−1^ once every 24 h for pain alleviation; their behavioral activity, food intake, excretion, and physical appearance in the home cage were assessed daily to ensure they remained in a healthy state. The virus injection locations were validated in each mouse after all experiments.

### Cannula Implantation And Intracranial Microinfusion

Mice were anesthetized using isoflurane and head was fixed in a stereotaxic device. A cannula was implanted above the target brain region for drug infusion and one stainless‐steel screws were implanted in the skull for support, all secured with dental cement. A stainless‐steel closure was inserted into each catheter to prevent occlusion. TIP39 (100 pm, 1 µL) was infused into each side of the dCA1 regions. The injector cannula was left in the dCA1 for additional 5 min to allow adequate local drug diffusion and minimize the spread of the drug along the injection track. Behavioral tests were performed at the indicated time.

### Statistical Analysis

All results presented in this study were from at least three independent experiments. GraphPad Prism 10 software was used for statistical analyses. For two groups of samples comparisons, student's *t*‐test or Mann‐Whitney U test was used to evaluate statistical significance. For multiple comparisons of three or more groups of samples, ANOVA was used. Statistical significance was defined as *p* < 0.05.

## Conflict of Interest

The authors declare no conflict of interest.

## Author Contributions

Z.‐J.L., J.‐R.B., Z.‐Y.Y., and M. T. contributed equally to this work. D.S., W.‐B.C., and X.‐J.Z. designed the project and wrote the manuscript; Z.‐J.L., J.‐R.B., Z.‐Y.Y., M.T., Z.‐Y.C., and R.W. performed CRS, CSDS, behavioral tests, virus injection, immunostaining, RNA scope, calcium recording, electrophysiological recordings and data analysis; M.‐M.W., H.‐W.Z., Y.‐Q.Z., H.‐J.W., B.‐Y.Q., and S.‐S. S. performed mouse genotyping, western blot and data quantification; D.S. helped data analysis and interpretation; D.S. supervised the project.

## Supporting information



Supporting Information

Supplemental Video 1

Supplemental Video 2

## Data Availability

The data that support the findings of this study are available from the corresponding author upon reasonable request.
