# Supplementary material for: Adenylyl Cyclase 8 in Dorsal CA1 Neurons Prevents Depressive‐Like Behaviors by Maintaining Neuronal Excitability and Glutamatergic Neurotransmission Through TIP39‐PTH2R Signaling
Source: Adv Sci (Weinh). 2025 Dec 12;13(10):e12170. doi: 10.1002/advs.202512170 (PMC12915128; doi:10.1002/advs.202512170)
Supplement: Supplementary file 1 — Supporting Information [file ADVS-13-e12170-s001.docx]

**Supplemental information**

**Adenylyl cyclase 8 in dorsal CA1 neurons prevents depressive-like behaviors by maintaining neuronal excitability and glutamatergic neurotransmission through TIP39-PTH2R signaling**

Zi-Jie Liu^1#^, Jia-Rui Bi^1#^, Zong-Yan Yu^2,3#^, Meng Tian^4#^, Zhi-Yue Chen^1^, Ran Wei^1^, Miao-Miao Wang^1^, Hai-Wei Zha^1^, Yu-Qing Zhang^1^, Hong-Jing Wang^2^, Bang-You Qiang^2^, Shuang-Shuang Sun^2,3^, Xiao-Juan Zhu^4*^, Wen-Bing Chen^2,3*^, Dong Sun^1,5*^

1 National Engineering Laboratory for AIDS Vaccine, School of Life Sciences, Jilin University, Changchun, China

2 The Affiliated TCM Hospital, School of Basic Medical Sciences, Guangzhou Medical University, Guangzhou, China

3 Key Laboratory of Neurogenetics and Channelopathies of Guangdong Province and the Ministry of Education of China, Guangzhou Medical University, Guangzhou, China

4 Key Laboratory of Molecular Epigenetics, Ministry of Education, Institute of Genetics and Cytology, Northeast Normal University, Changchun, China

5 Key Laboratory for Molecular Enzymology and Engineering, The Ministry of Education, School of Life Sciences, Jilin University, Changchun, China.

^#^ These authors contribute equally

*Corresponding author: [dongsun@jlu.edu.cn](mailto:dongsun@jlu.edu.cn); [chenwenbing@gzhmu.edu.cn](mailto:chenwenbing@gzhmu.edu.cn); [zhuxj720@nenu.edu.cn](mailto:zhuxj720@nenu.edu.cn)

**Figure S1. CRS induced depressive- and anxiety-like behaviors in mice**


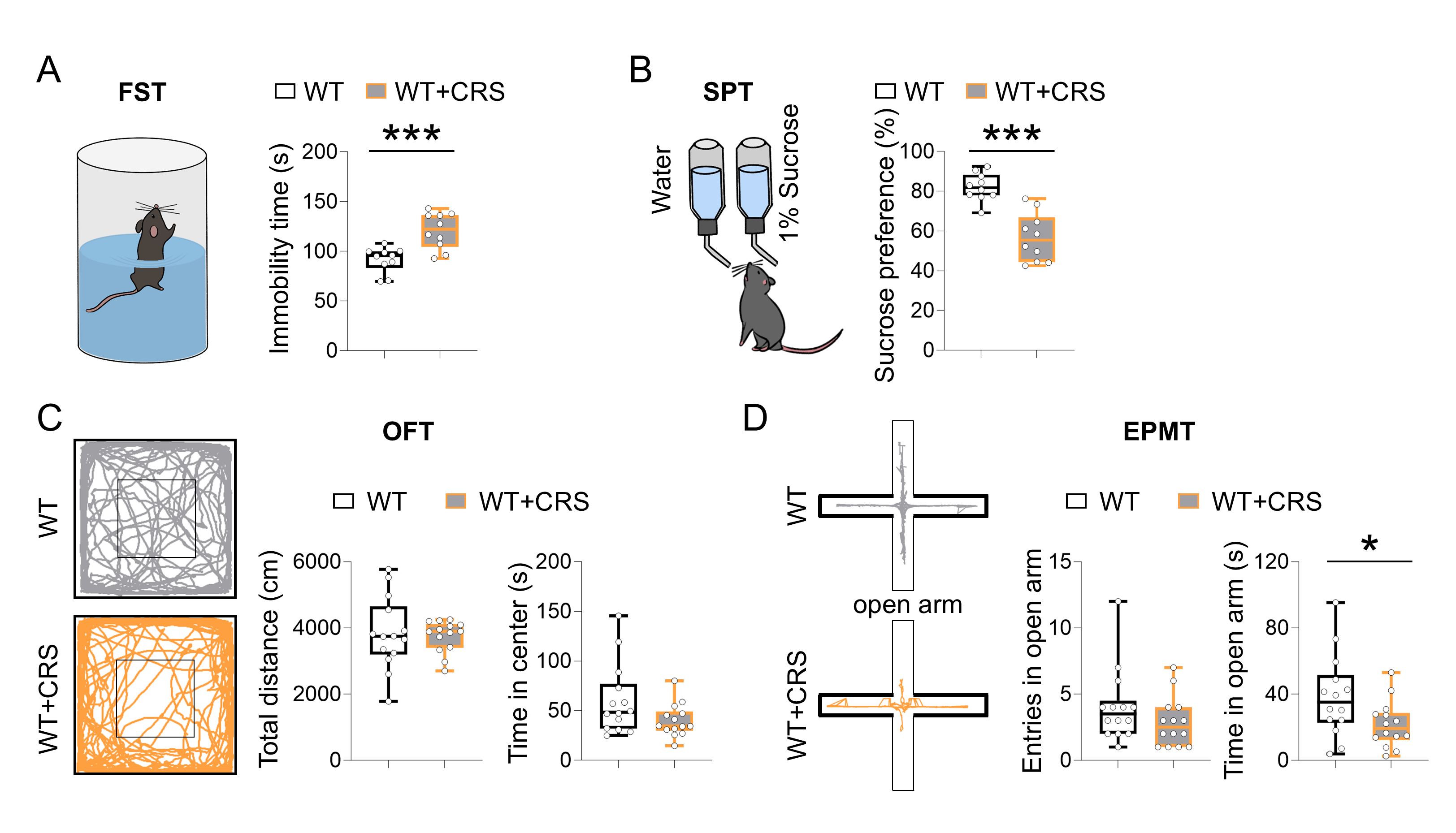


(A) Schematic diagram of FST and quantification of immobility time in the FST of WT and WT+CRS mice. n = 10 mice for each group. Student’s t test, p < 0.001.

(B) Schematic diagram of SPT and quantification of sucrose preference in the SPT of WT and WT+CRS mice. n = 10 mice for each group. Student’s t test, p < 0.001.

(C) Representative tracing images and quantifications of total distance and center duration time in the OFT of WT and WT+CRS mice. n = 14 mice for each group. Mann-Whitney U test for total distance, p = 0.7345; Mann-Whitney U test for time in center, p = 0.1251.

(D) Representative tracing images and quantifications of open arm duration time and entries in the EPMT of WT and WT+CRS mice. n = 14 mice for each group. Mann-Whitney U test for entries in open arm, p = 0.1704; Student’s t test for time in open arm, p = 0.036.

Data are presented as median with interquartile range; whiskers are the minimum and maximum. *p < 0.05, ***p < 0.001.

**Figure S2. 1 day of ARS did not change *Adcy8* expression in the hippocampus**


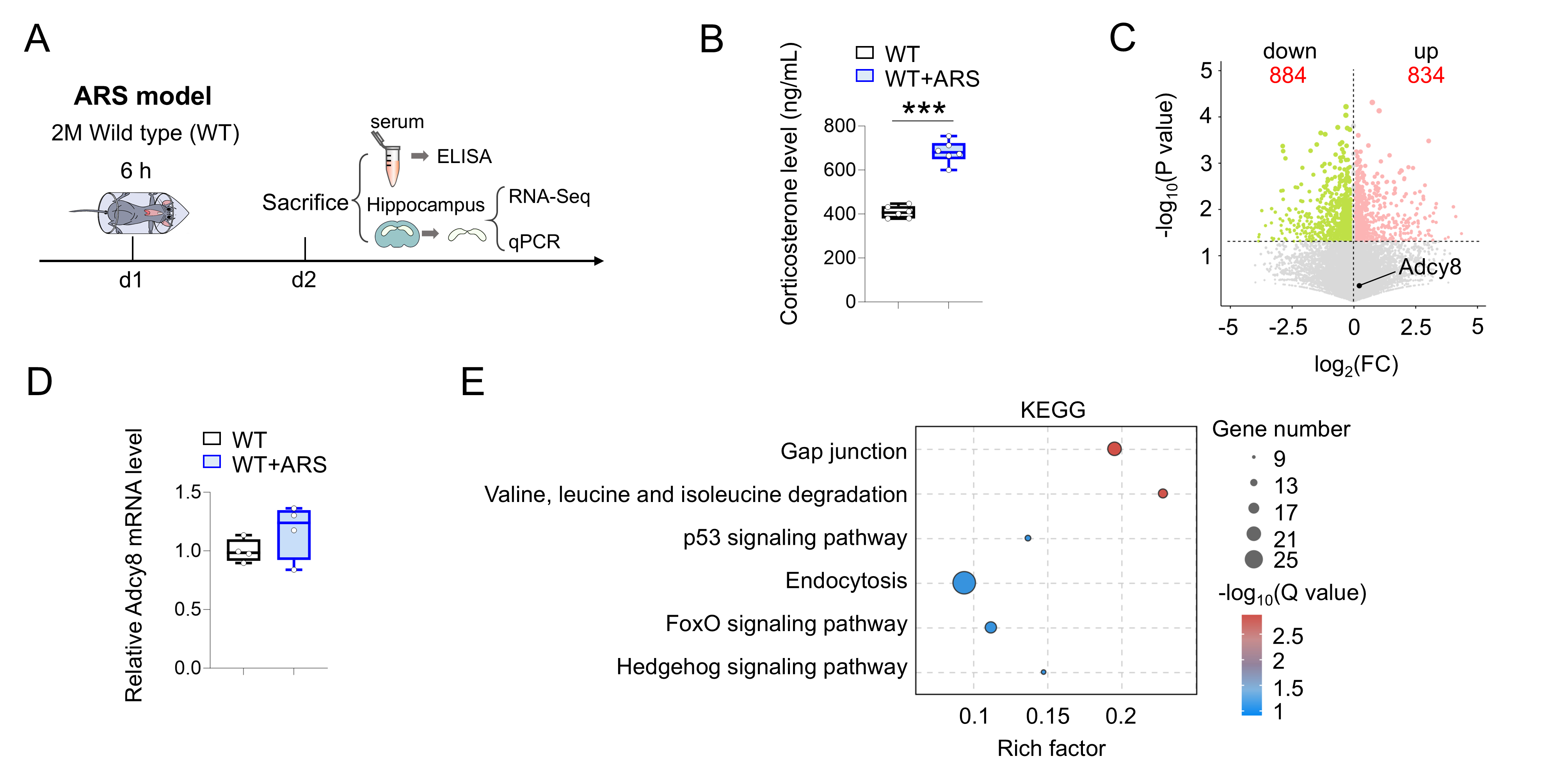


(A) Schematic diagram of experimental design for ELISA, RNA-seq and qPCR analyses in WT and WT+ARS mice.

(B) ELISA analysis of corticosterone in the plasma of WT and WT+ARS mice. n = 6 mice for each group. Student’s t test. p < 0.001.

(C) Volcano plots of differentially expressed genes in the WT+ARS hippocampus as compared to WT hippocampus.

(D) qPCR analysis of *Adcy8* mRNA levels in the hippocampus of WT and WT+ARS mice. n = 4 mice for each group. Student’s t test. p = 0.2288.

(E) KEGG analysis of the dysregulated genes in C.

Data in B and D are presented as median with interquartile range; whiskers are the minimum and maximum. ***p < 0.001.

**Figure S3. CRS had little effect on *Adcy8* expression in the other hippocampal subregions**


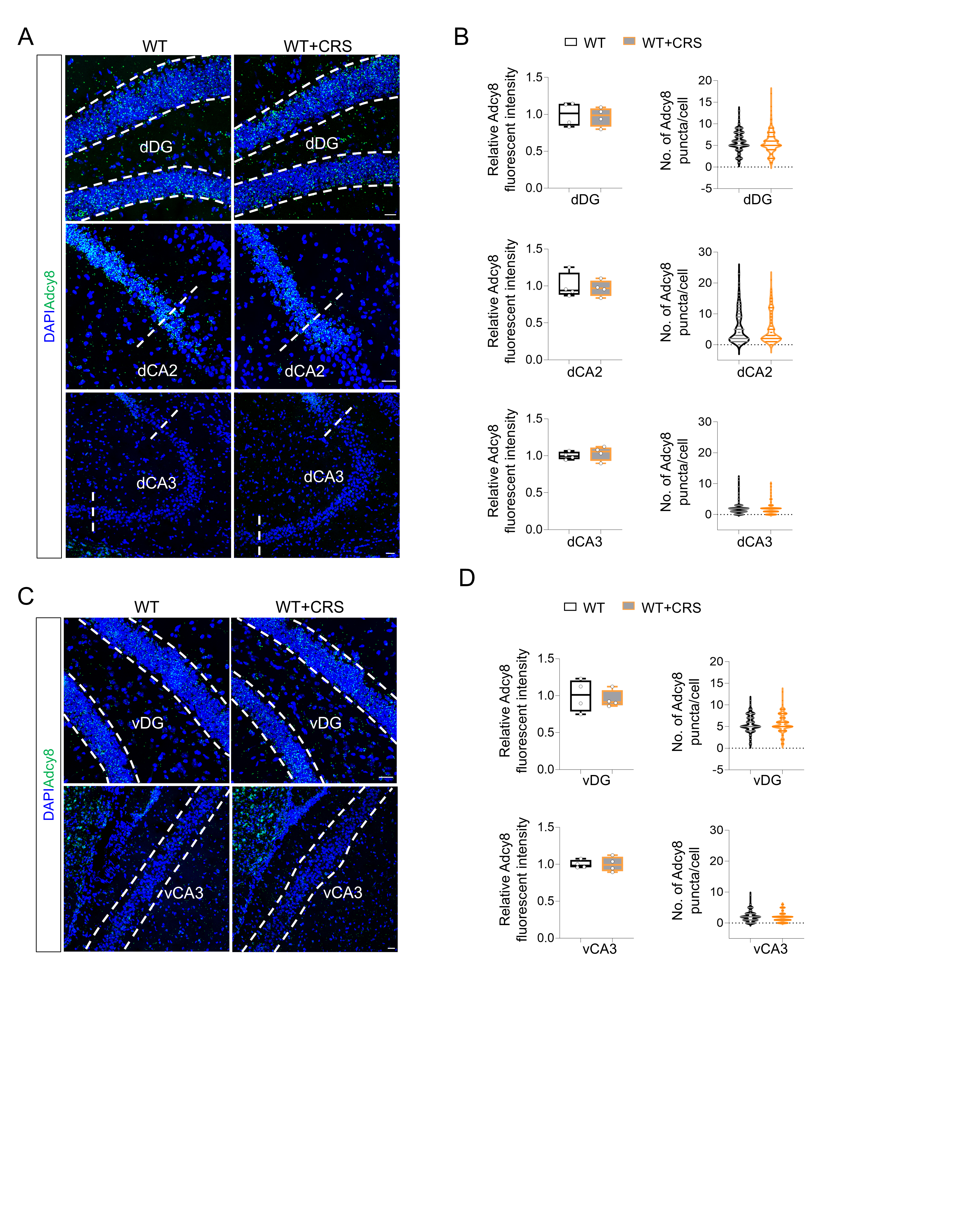


(A) Representative RNA-scope images of *Adcy8* in dDG, dCA2 and dCA3 subregions of WT and WT+CRS mice. Scale bar = 20 μm.

(B) Quantifications of the data in A, the fluorescent intensity of *Adcy8* mRNA and the number of *Adcy8* puncta surrounding cell nucleus in WT and WT+CRS mice. n = 4 mice for each group. Student’s t test for *Adcy8* fluorescent intensity comparison. dDG: p = 0.7595, dCA2: p = 0.7642; dCA3: p = 0.5821. Mann-Whitney U test for *Adcy8* puncta comparison. dDG: p = 0.0843, dCA2: p = 0.8822; dCA3: p = 0.6584.

(C) Representative RNA-scope images of *Adcy8* in vDG and vCA3 subregions of WT and WT+CRS mice. Scale bar = 20 μm.

(D) Quantifications of the data in C, the fluorescent intensity of *Adcy8* mRNA and the number of *Adcy8* puncta surrounding cell nucleus in WT and WT+CRS mice. n = 4 mice for each group. Student’s t test for *Adcy8* fluorescent intensity comparison. vDG: p = 0.7099, vCA3: p = 0.9973. Mann-Whitney U test for *Adcy8* puncta comparison. vDG: p = 0.5631; vCA3: p = 0.0803.

Data of fluorescent intensity of *Adcy8* mRNA are presented as median with interquartile range, whiskers are the minimum and maximum; quantifications of *Adcy8* puncta are presented as violin plot with all points.

**Figure S4. CSDS susceptible mice showed reduced *Adcy8* mRNAs in dCA1 neurons**


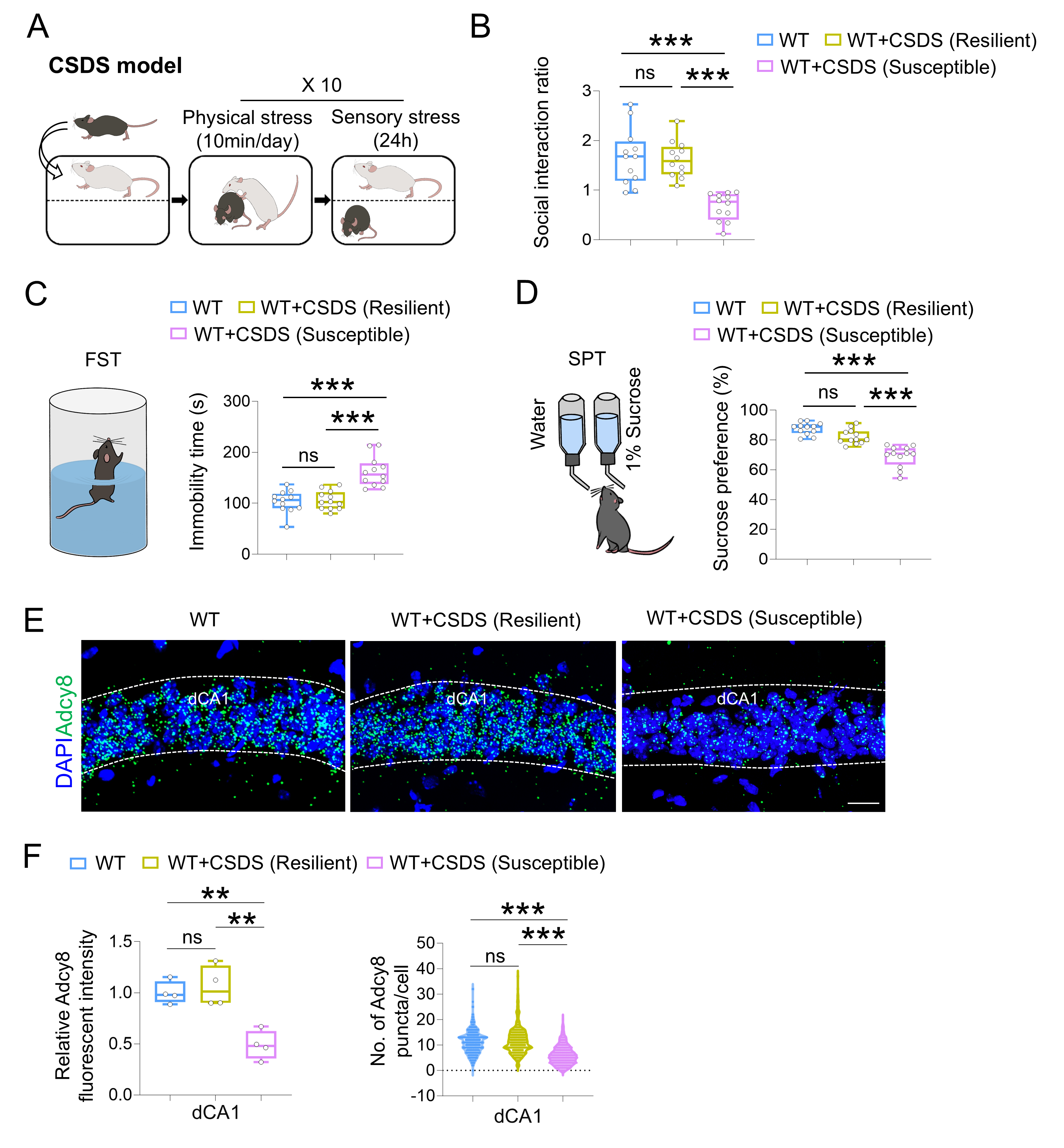


(A) Schematic diagram of experimental design for CSDS model.

(B) CSDS treated mice were divided into susceptible and resilient groups by their social interaction ratio. n = 12 mice for each group. One-way ANOVA followed by Tukey’s multiple comparisons test. F_(2, 33)_ = 21.20, p < 0.001. WT vs WT+CSDS (resilient), p = 0.9564; WT vs WT+CSDS (susceptible), p < 0.001; WT+CSDS (resilient) vs WT+CSDS (susceptible), p < 0.001.

(C) Schematic diagram of FST and quantification of immobility time in the FST of WT, WT+CSDS (resilient) and WT+CSDS (susceptible) mice. n = 12 mice for each group. One-way ANOVA followed by Tukey’s multiple comparisons test. F_(2, 33)_ = 23.55, p < 0.001. WT vs WT+CSDS (resilient), p = 0.9579; WT vs WT+CSDS (susceptible), p < 0.001; WT+CSDS (resilient) vs WT+CSDS (susceptible), p < 0.001.

(D) Schematic diagram of SPT and quantification of sucrose preference in the SPT of WT and WT+CSDS mice. n = 12 mice for each group. One-way ANOVA followed by Tukey’s multiple comparisons test. F_(2, 33)_ = 35.44, p < 0.001. WT vs WT+CSDS (resilient), p = 0.0571; WT vs WT+CSDS (susceptible), p < 0.001; WT+CSDS (resilient) vs WT+CSDS (susceptible), p < 0.001.

(E) Representative RNA-scope images of *Adcy8* in dCA1 regions of WT, WT+CSDS (resilient) and WT+CSDS (susceptible) mice. Scale bar = 20 μm.

(F) Quantifications of the data in E, the fluorescent intensity of *Adcy8* mRNA and the number of *Adcy8* puncta surrounding cell nucleus. n = 4 mice for each group. One-way ANOVA followed by Tukey’s multiple comparisons test. For fluorescent intensity: F_(2, 9)_ = 16.36, p = 0.001. WT vs WT+CSDS (resilient), p = 0.8533; WT vs WT+CSDS (susceptible), p = 0.003; WT+CSDS (resilient) vs WT+CSDS (susceptible), p = 0.0015. For *Adcy8* puncta: F_(2, 1377)_ = 251.7, p < 0.001. WT vs WT+CSDS (resilient), p = 0.1212; WT vs WT+CSDS (susceptible), p < 0.001; WT+CSDS (resilient) vs WT+CSDS (susceptible), p < 0.001.

Data in B-D, F are presented as median with interquartile range; whiskers are the minimum and maximum. **p < 0.01, ***p < 0.001, ns = no significance.

**Figure S5 CRS decreased *Adcy8* mRNAs in CaMKII^+^ dCA1 neurons**

**
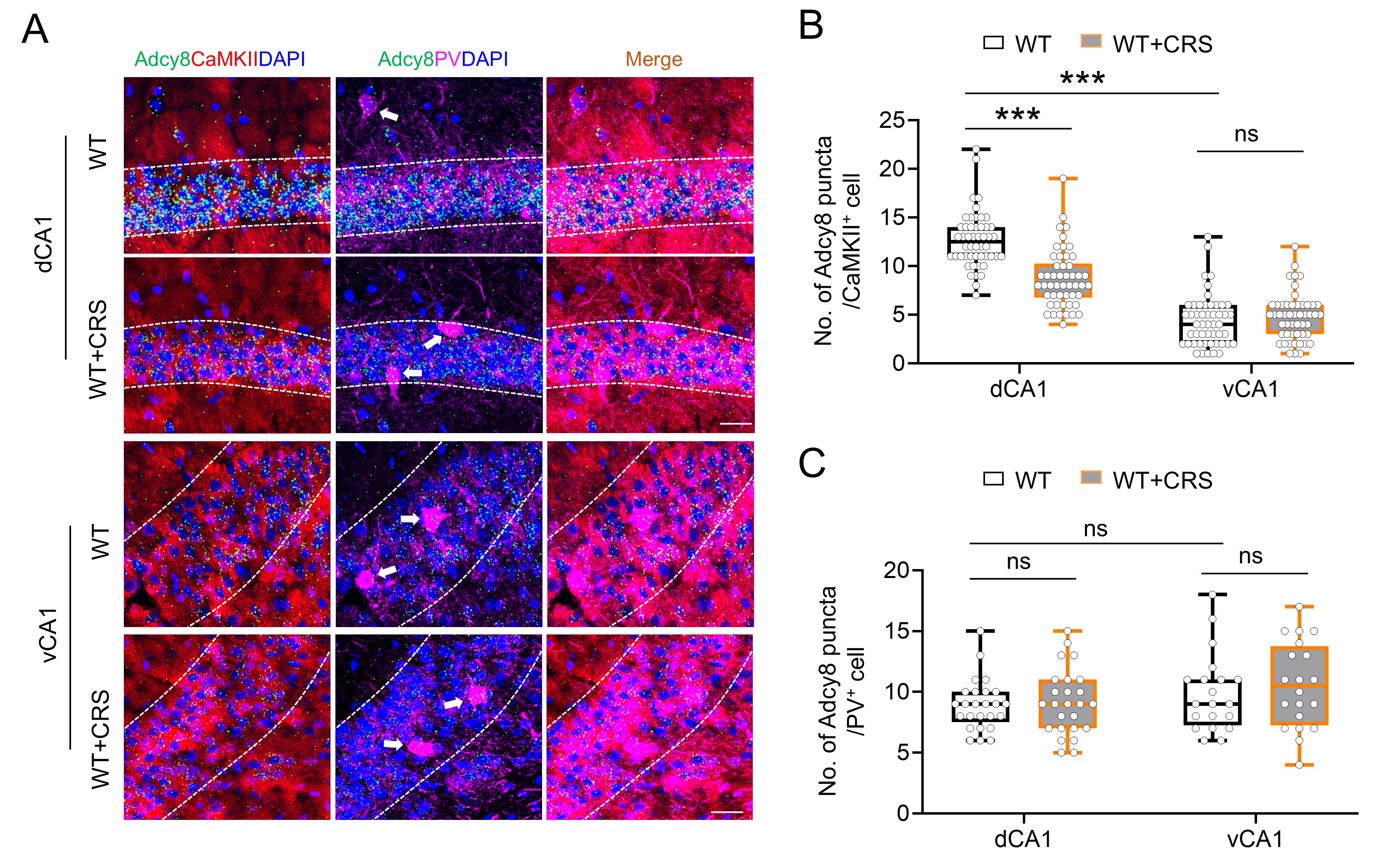
**

(A) Co-detection of *Adcy8* mRNAs (green) in CamKII^+^ (red) excitatory neurons and PV^+^ (purple) inhibitory neurons in the dCA1 and vCA1 regions. DAPI (blue) was stained for cell nucleus. Scale bar = 20 μm.

(B) Quantifications of the data in A, the number of *Adcy8* puncta per CaMKII^+^ cells. n = 4 mice for each group. Two-way ANOVA followed by Tukey’s multiple comparisons test. Interaction: F_(1, 196)_ = 31.89, p < 0.001; treatment factor: F_(1, 196)_ = 20.83, p < 0.001; region factor: F_(1, 196)_ = 249.8, p < 0.001. For dCA1 region: WT vs WT+CRS, p < 0.001; For vCA1 region: WT vs WT+CRS, p = 0.9707; For WT mice, dCA1 vs vCA1, p <0.001.

(C) Quantifications of the data in A, the number of *Adcy8* puncta per PV^+^ cells. n = 4 mice for each group. Two-way ANOVA followed by Tukey’s multiple comparisons test. Interaction: F_(1, 86)_ = 0.1112, p = 0.7395; treatment factor: F_(1, 86)_ = 0.5242, p = 0.4710; region factor: F_(1, 86)_ = 3.717, p = 0.0572. For dCA1 region: WT vs WT+CRS, p = 0.9999; For vCA1 region: WT vs WT+CRS, p = 0.9802; For WT mice, dCA1 vs vCA1, p = 0.8393.

Data in B and C are presented as median with interquartile range; whiskers are the minimum and maximum. ***p < 0.001, ns = no significance.

**Figure S6 Global KO of *Adcy8* increased anxiety-like behaviors, whereas CKO of *Adcy8* in CaMKII^+^ neurons had little effect on anxiety-like behaviors in mice**


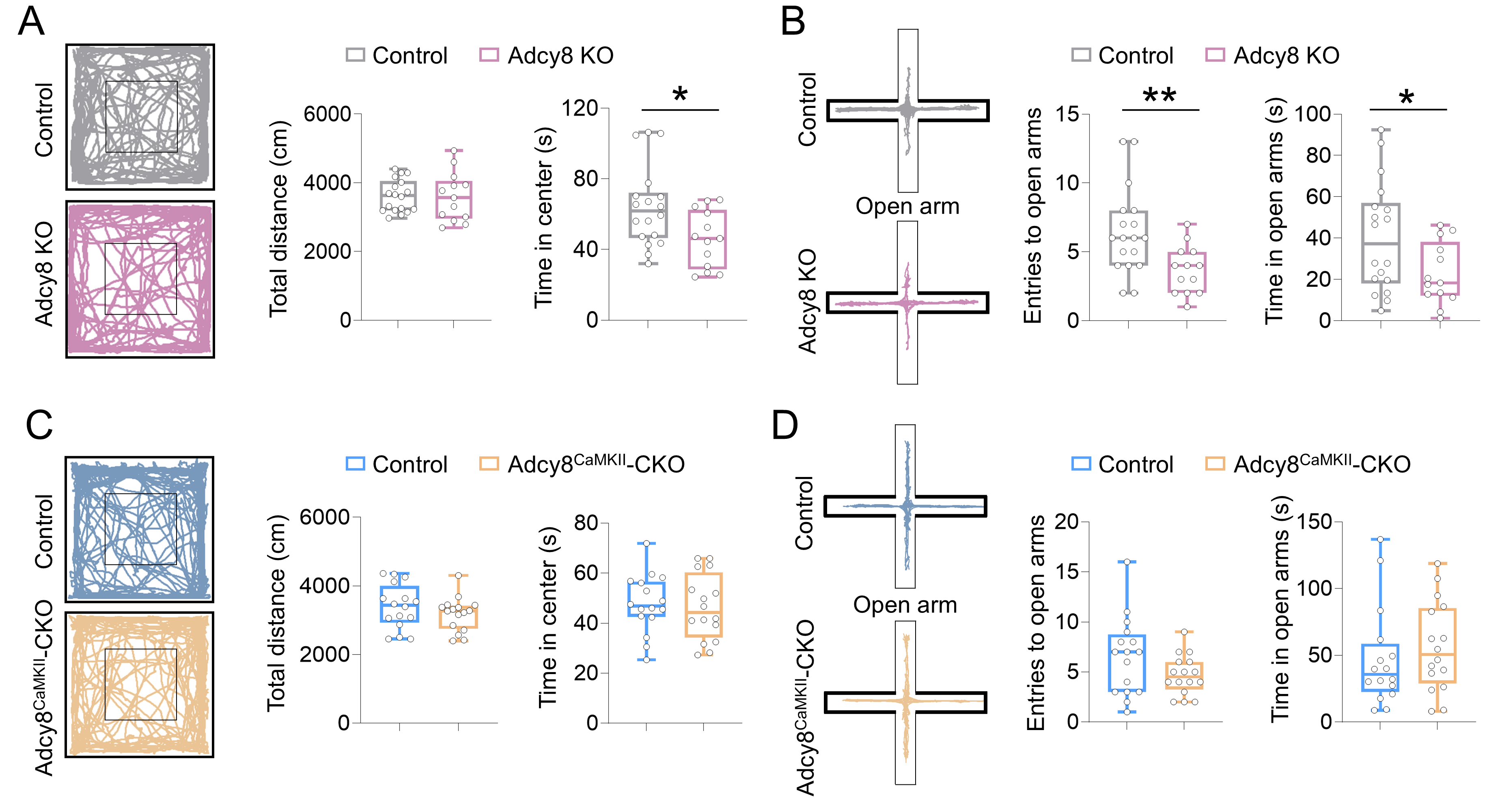


(A) Representative tracing images and quantifications of total distance and center duration time in the OFT of control (n = 18) and *Adcy8* KO (n = 13) mice. Student’s t test for total distance, p = 0.9015; Student’s t test for time in center, p = 0.0193.

(B) Representative tracing images and quantifications of open arm duration time and entries in the EPMT of control (n = 18) and Adcy8 KO (n = 13) mice. Student’s t test for entries to open arms, p = 0.0078; Student’s t test for time in open arms, p = 0.0404.

(C) Representative tracing images and quantifications of total distance and center duration time in the OFT of control and Adcy8^CaMKII^-CKO mice. n = 16 mice for each group. Student’s t test for total distance, p = 0.2947; Student’s t test for time in center, p = 0.7585.

(D) Representative tracing images and quantifications of open arm duration time and entries in the EPMT of control and Adcy8^CaMKII^-CKO mice. n = 16 mice for each group. Student’s t test for entries to open arms, p = 0.0969; Mann-Whitney U test for time in open arms, p = 0.3608.

Data are presented as median with interquartile range; whiskers are the minimum and maximum. *p < 0.05, **p < 0.01.

**Figure S7 *Adcy8* CKO in PV^+^ inhibitory neurons had little effect on depressive- and anxiety-like behaviors**


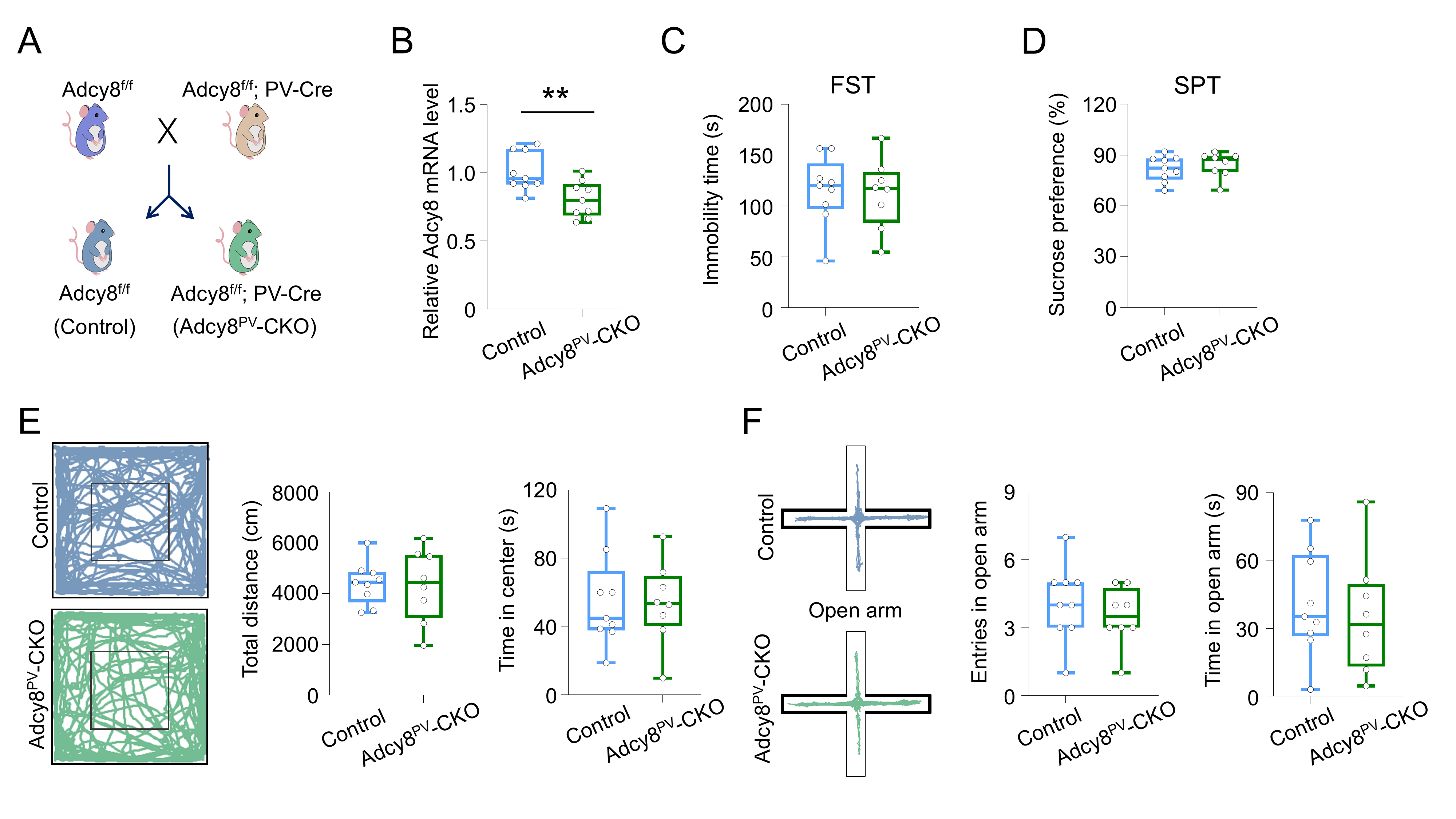


(A) Schematic diagram of breeding strategy to generate Adcy8^PV^-CKO mice. Adcy8^f/f^ mice are control.

(B) qPCR analysis of *Adcy8* mRNA levels in the hippocampus of control and Adcy8^PV^-CKO mice. n = 9 mice for each group. Student’s t test, p = 0.0063.

(C) Quantifications of immobility time in the FST of control (n = 9) and Adcy8^PV^-CKO (n = 8) mice. Student’s t test, p = 0.8375.

(D) Quantifications of sucrose preference in the SPT of control (n = 9) and Adcy8^PV^-CKO (n = 8) mice. Student’s t test, p =0.5134.

(E) Representative tracing images and quantifications of total distance and center duration time in the OFT of control (n = 9) and Adcy8^PV^-CKO (n = 8) mice. Student’s t test for total distance, p = 0.8922; Student’s t test for time in center, p = 0.9171.

(F) Representative tracing images and quantifications of open arm duration time and entries in the EPMT of control (n = 9) and Adcy8^PV^-CKO (n = 8) mice. Student’s t test for entries to open arms, p = 0.4225; Student’s t test for time in open arms, p = 0.6217.

Data in B-F are presented as median with interquartile range; whiskers are the minimum and maximum. **p < 0.01.

**Figure S8 Adcy8^CaMKII^-CKO mice exhibited deficits in struggling behavior with calcium transients**


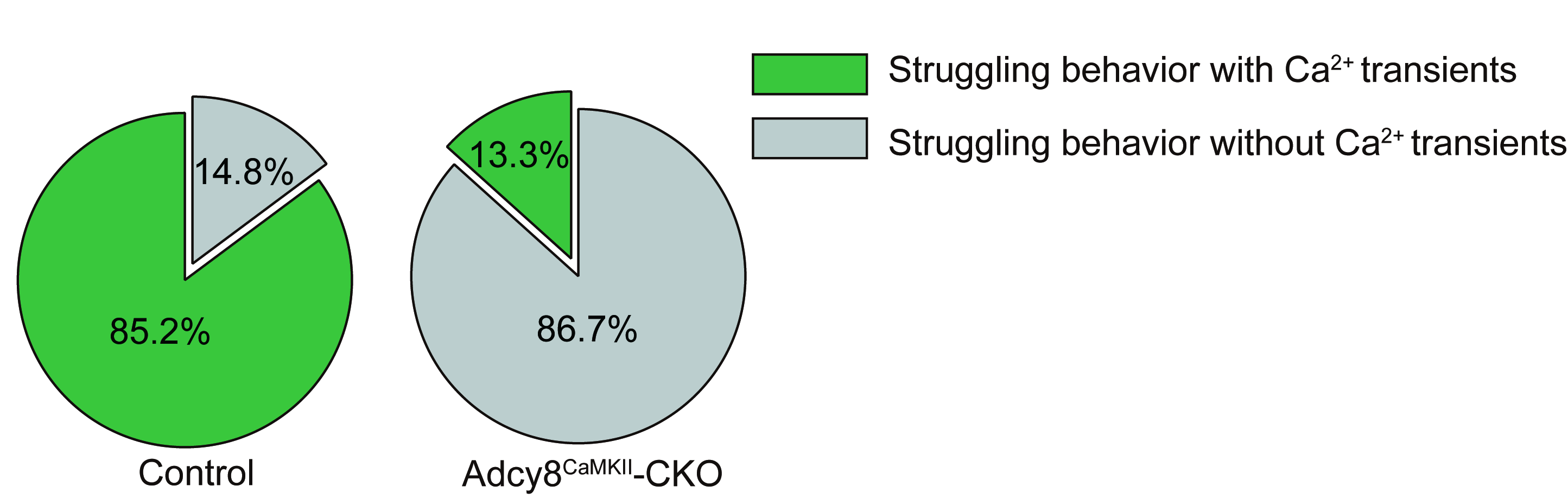


**Figure S9. Thy1-GFP labels excitatory neurons in the mouse brain**


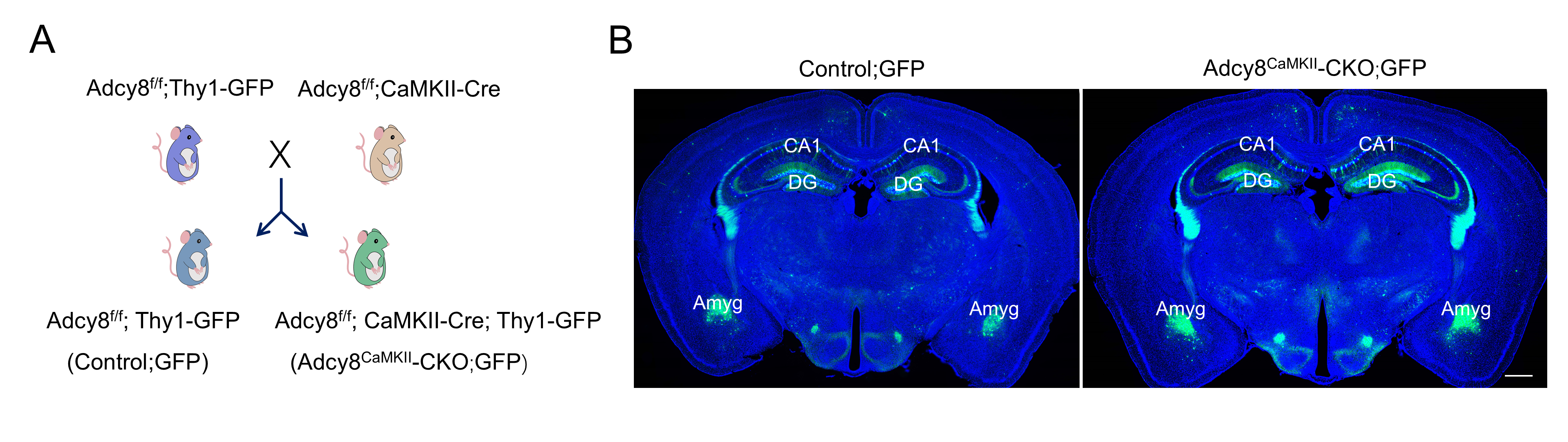


(A) Schematic diagram of breeding strategy to generate Adcy8^f/f^; Thy1-GFP (Control; GFP) mice and Adcy8^f/f^; CaMKII-Cre; Thy1-GFP (Adcy8^CaMKII^-CKO; GFP) mice.

(B) Representative images indicate that Thy1-GFP signal randomly labels excitatory neurons in multiple brain regions. Amyg stands for amygdala.

**Figure S10. Reduced hippocampal cAMP levels in Adcy8^CaMKII^-CKO and CRS treated mice**


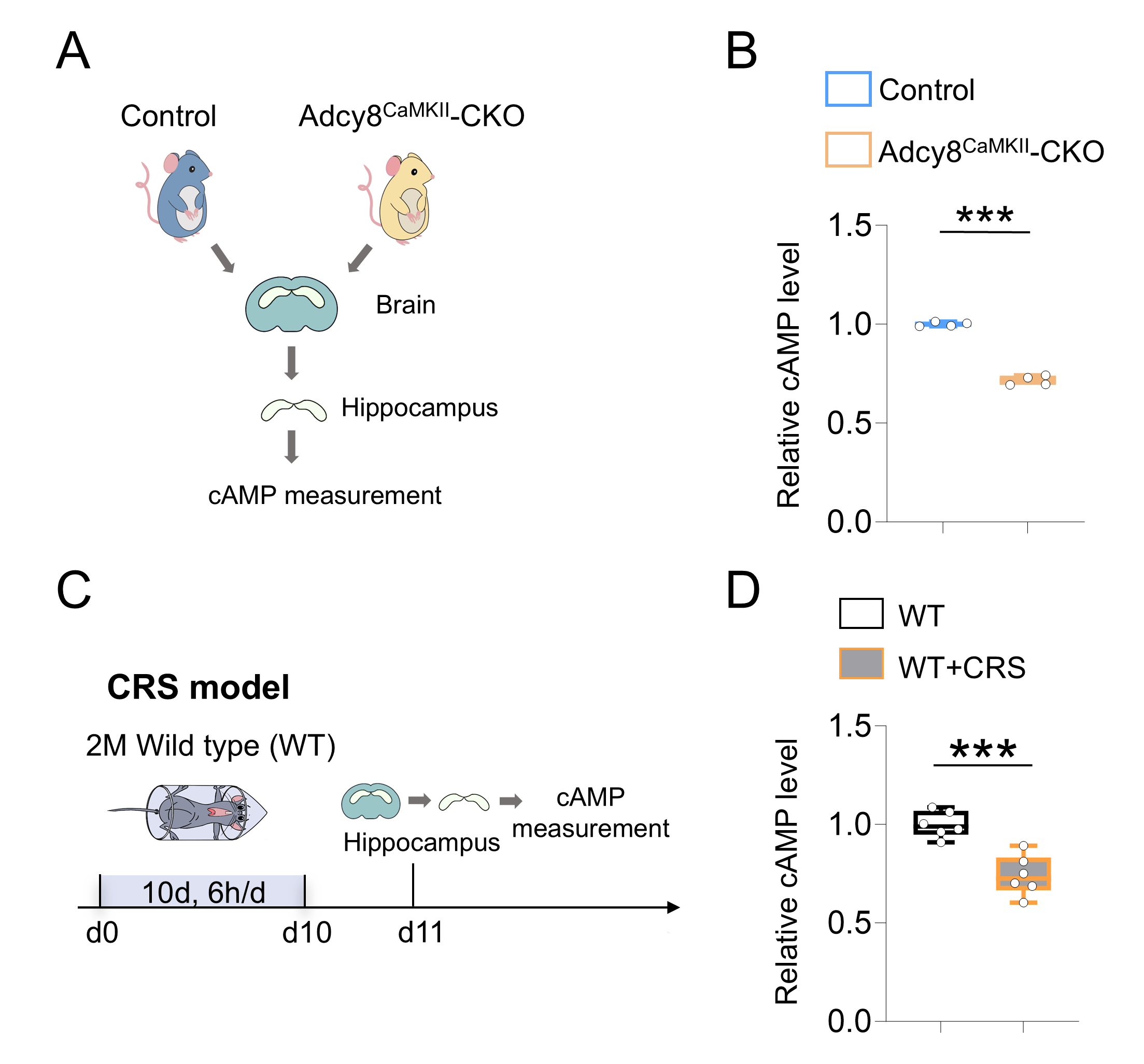


(A) Schematic diagram of cAMP measurement by ELISA analysis in control and Adcy8^CaMKII^-CKO hippocampus.

(B) Quantification of the cAMP level in A. n = 4 mice for each group. Student’s t test, p < 0.001.

(C) Schematic diagram of cAMP measurement by ELISA analysis in WT and WT+CRS hippocampus.

(D) Quantification of the cAMP level in C. n = 6 mice for each group. Student’s t test, p < 0.001.

Data in B and D are presented as median with interquartile range; whiskers are the minimum and maximum. ***p < 0.001.

**Figure S11. Comparable levels of MAPK signaling pathway between control and Adcy8^PV^-CKO mice**


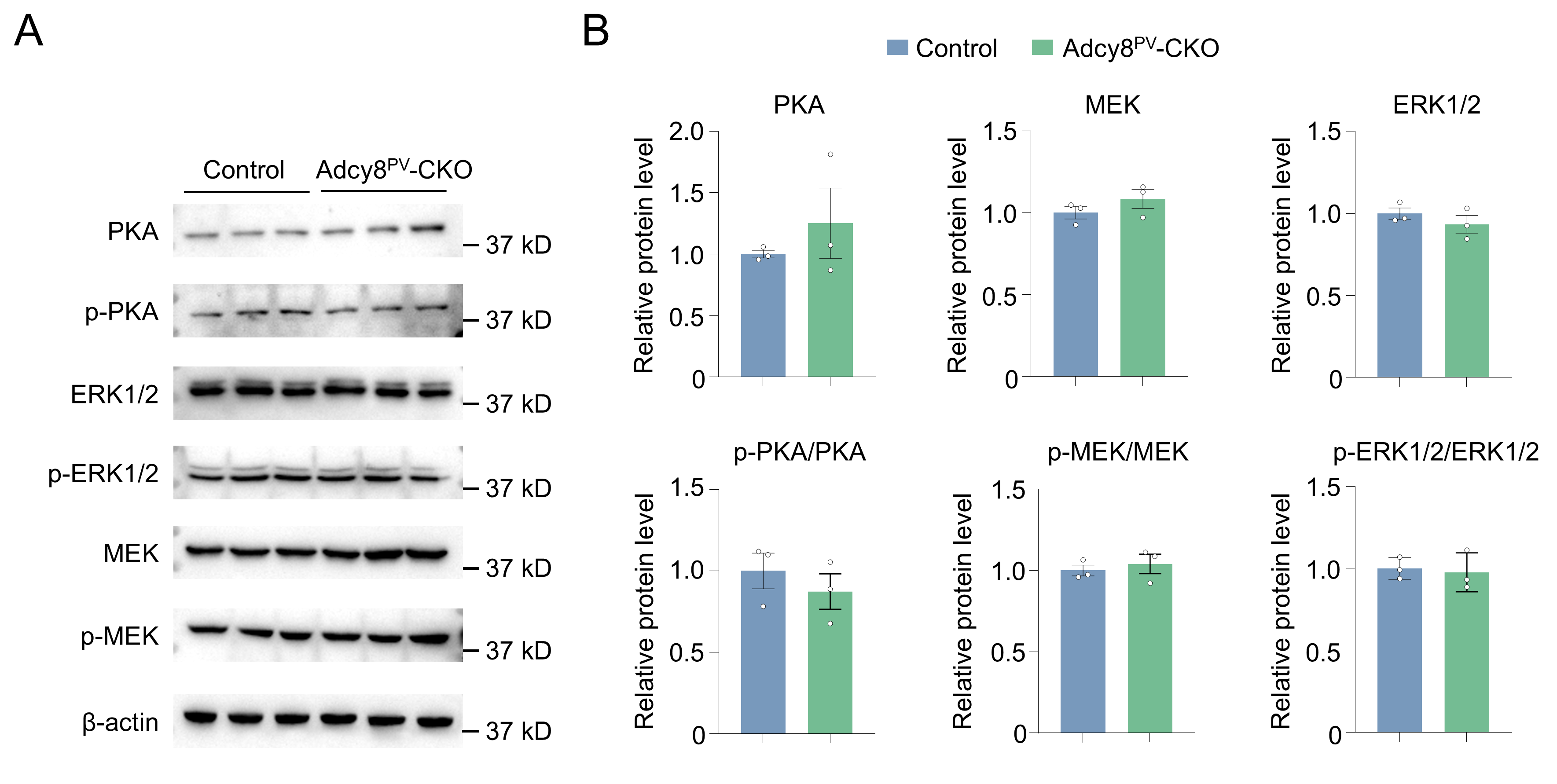


(A) Western blot analyses of lysates in the hippocampus of control and Adcy8^PV^-CKO mice.

(B) Quantifications of the data in A, the relative band intensity of PKA, p-PKA, MEK, p-MEK, ERK1/2 and p-ERK1/2. n = 3 mice for each group. Student’s t test. PKA, p = 0.4313; MEK, p = 0.2862; ERK1/2, p = 0.3681; p-PKA/PKA, p = 0.4568; p-MEK/MEK, p = 0.5871; p-ERK1/2/ERK1/2, p = 0.7843.

Data in B is presented as the mean ± SEM.

**Figure S12. CRS decreased PTH2R expression and downregulated MAPK signaling**


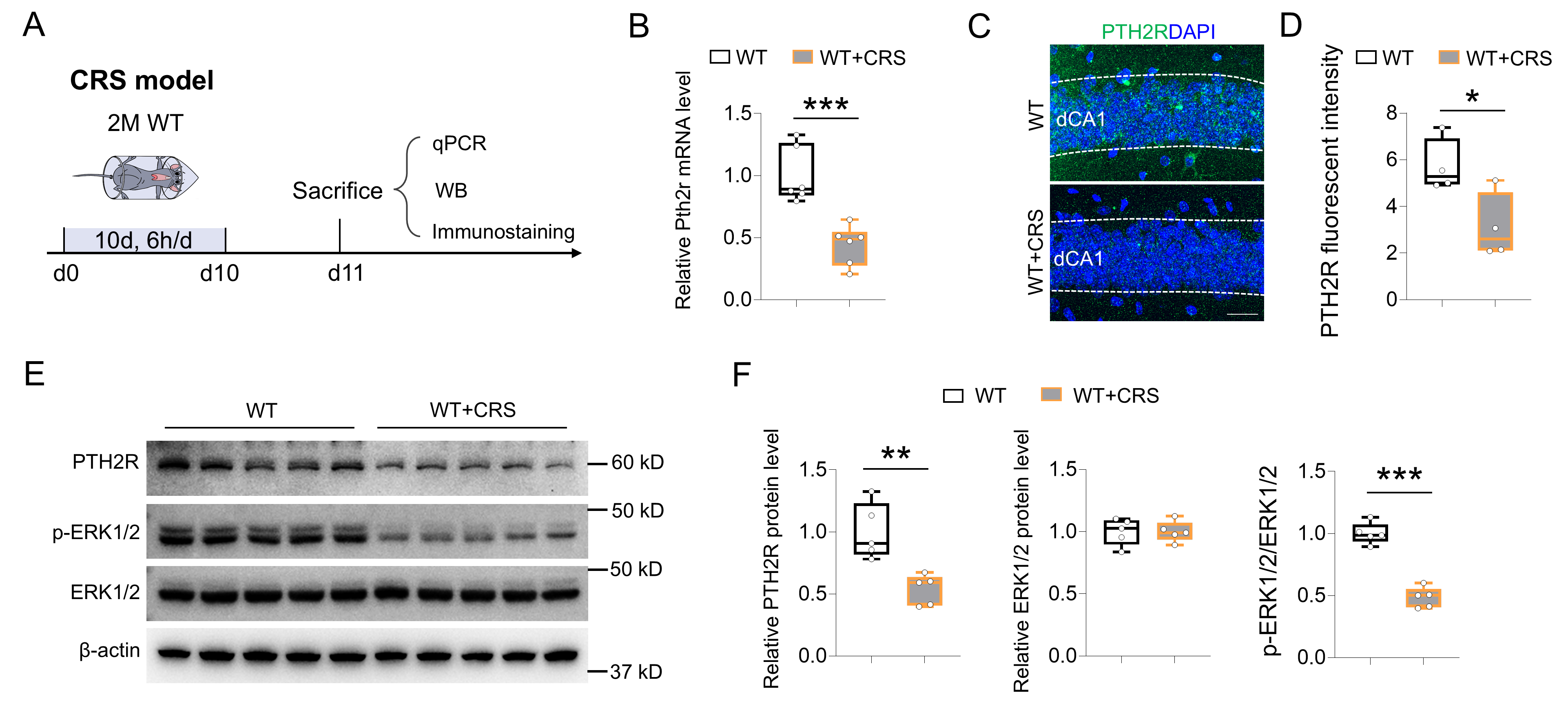


(A) Schematic diagram of qPCR, WB and immunostaining analyses in WT and WT+CRS hippocampus.

(B) qPCR analysis of *Pth2r* mRNA levels in the hippocampus of WT and WT+CRS mice. n = 6 mice for each group. Student’s t test, p < 0.001.

(C) Immunostaining of PTH2R (green) in the dCA1 regions of WT and WT+CRS mice. DAPI (blue) was stained for cell nucleus. Scale bar = 20 μm.

(D) Quantification of the data in C, the fluorescent intensity of PTH2R. n = 4 mice for each group. Student’s t test, p = 0.0285.

(E) Western blot analysis of PTH2R, p-ERK1/2 and ERK1/2 in the hippocampus of WT and WT+CRS mice.

(F) Quantification of the data in E. n = 5 mice for each group. Student’s t test. PTH2R, p = 0.0037; ERK1/2, p = 0.9822; pERK1/2/ERK1/2, p < 0.001.

Data in B, D and F are presented as median with interquartile range; whiskers are the minimum and maximum. *p < 0.05, **p < 0.01, ***p < 0.001.

**Figure S13 Knockdown of *Pth2r* in the hippocampus had little effect on anxiety-like behaviors**


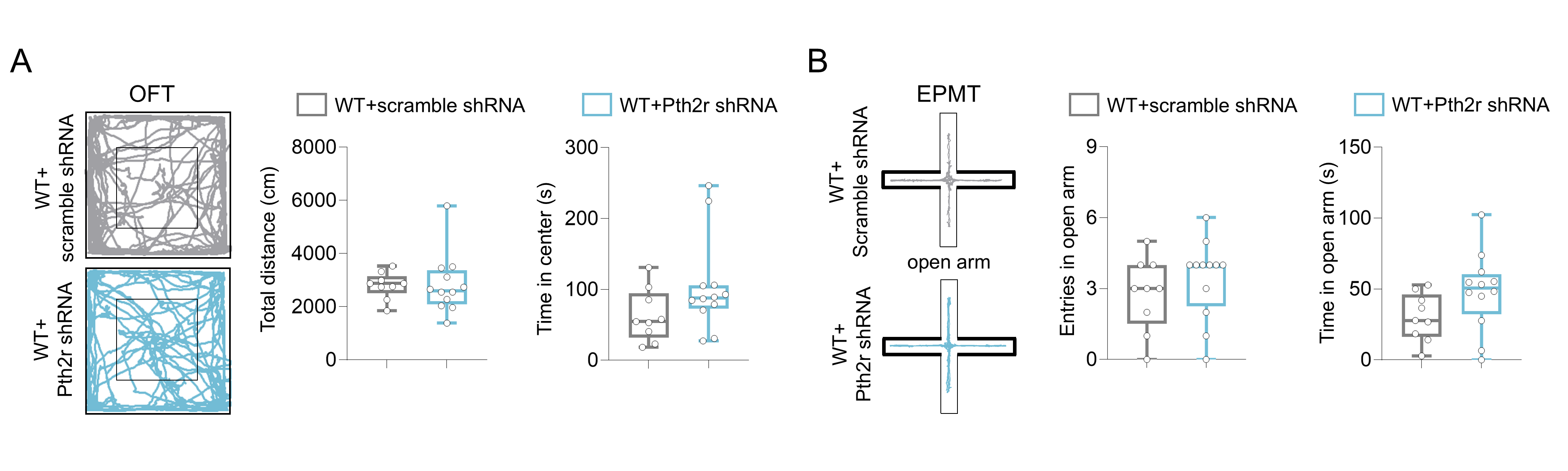


(A) Representative tracing images and quantifications of total distance and center duration time in the OFT of WT+scramble shRNA (n = 9) and WT+Pth2r shRNA (n = 12) mice. Mann-Whitney U test for total distance, p = 0.5079; Mann-Whitney U test for time in center, p = 0.1111.

(B) Representative tracing images and quantifications of open arm duration time and entries in the EPMT of WT+scramble shRNA mice (n = 9) and WT+Pth2r shRNA (n = 12) mice. Student’s t test for entries in open arm, p = 0.3851; Student’s t test for time in open arm, p = 0.1023.

Data in A and B are presented as median with interquartile range; whiskers are the minimum and maximum.

**Figure S14 Infusion of TIP39 in the dCA1 regions reversed the depressive-like behaviors in Adcy8^CaMKII^-CKO mice**


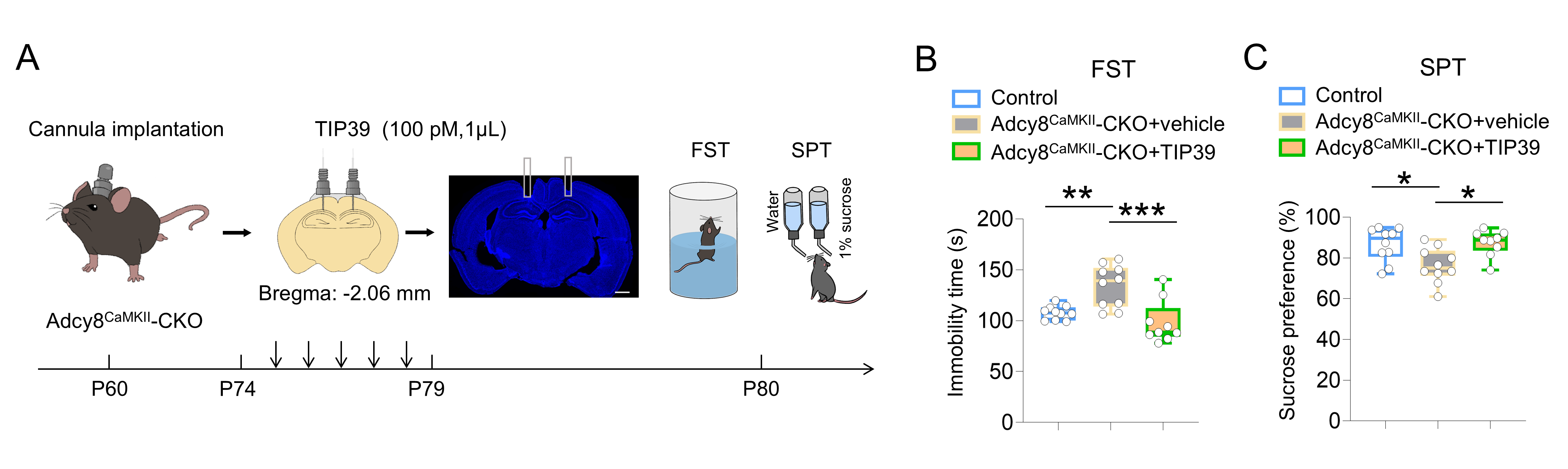


(A) Schematic diagram of experimental design for behavioral tests in Adcy8^CaMKII^-CKO mice injected with 100 pM TIP39 or vehicle. Adcy8^f/f^ mice are controls.

(B) Quantifications of immobility time in the FST of control (n = 10), Adcy8^CaMKII^-CKO+vehicle (n = 10) and Adcy8^CaMKII^-CKO+TIP39 (n = 9) mice. One-way ANOVA followed by Tukey’s multiple comparisons test. Interaction: F_(2, 26)_ = 11.21, p < 0.001. Control vs Adcy8^CaMKII^-CKO+vehicle, p = 0.006; Adcy8^CaMKII^-CKO+vehicle vs Adcy8^CaMKII^-CKO+TIP39, p < 0.001.

(C) Quantifications of sucrose preference in the SPT of control (n = 10), Adcy8^CaMKII^-CKO+vehicle (n = 10) and Adcy8^CaMKII^-CKO+TIP39 (n = 9) mice. One-way ANOVA followed by Tukey’s multiple comparisons test. Interaction: F_(2, 26)_ = 6.144, p = 0.0065. Control vs Adcy8^CaMKII^-CKO+vehicle, p = 0.0174; Adcy8^CaMKII^-CKO+vehicle vs Adcy8^CaMKII^-CKO+TIP39, p = 0.0128.

Data in B and C are presented as median with interquartile range; whiskers are the minimum and maximum. *p < 0.05, **p < 0.01, ***p < 0.001
